# Cross-national predictive correlates of depressive symptoms among middle-aged and older adults with chronic conditions: an explainable machine learning analysis

**DOI:** 10.3389/fpsyt.2026.1901045

**Published:** 2026-07-28

**Authors:** Xiaoyu Guo, Hanshuo Xing, Changhong Li, Di Wang, Xinhua Li, Xuanping Zhang, Yamin Liu, Yanfen She

**Affiliations:** 1 College of Acupuncture and Moxibustion, Hebei University of Chinese Medicine, Shijiazhuang, Hebei, China; 2 National Key Laboratory of Chinese Medicine Modernization, Haihe Laboratory of Modern Chinese Medicine, Tianjin University of Traditional Chinese Medicine, Tianjin, China; 3 College of Teacher Education, Guangdong University of Education, Guangzhou, Guangdong, China; 4 Hebei International Joint Research Center for Dominant Diseases in Chinese Medicine and Acupuncture, Shijiazhuang, Hebei, China; 5 School of Chinese Medicine, Hebei University of Chinese Medicine, Shijiazhuang, Hebei, China

**Keywords:** cross-national comparison, depressive symptoms, explainable machine learning, older adults, self-rated health

## Abstract

**Background:**

Rapid population aging has made depression among middle-aged and older adults an increasingly important public health concern. Although numerous risk factors have been identified, an integrative framework for clarifying how these risks are structured across sociocultural contexts remains lacking. Identifying cross-nationally stable vulnerability signals is therefore important for improving screening and prevention. This study aimed to identify cross-nationally stable and context-specific predictive correlates of depressive symptoms among middle-aged and older adults with at least one chronic condition using an explainable machine-learning framework.

**Methods:**

Data were drawn from six international longitudinal cohorts—CHARLS, ELSA, HRS, KLoSA, MHAS, and SHARE—including 84,140 participants aged 50 years or older who reported at least one chronic condition. Within an explainable artificial intelligence framework, six predictive algorithms were evaluated: LR, RF, SVM, MLP, XGBoost, and EBM. SMOTE was used to address class imbalance, and SHAP analysis was applied to characterize the hierarchical structure of risk determinants.

**Results:**

The models achieved moderate predictive performance across cohorts, with area under the receiver operating characteristic curve values ranging from 0.77 to 0.80. Explainable boosting machine and logistic regression performed nearly identically in most cohorts, with absolute AUC differences below 0.005 in five of six cohorts. A set of cross-nationally stable predictive correlates was identified, among which poor self-rated health, pain burden, and limitations in activities of daily living and instrumental activities of daily living were the most influential predictors. Conversely, demographic and socioeconomic factors showed greater cross-national heterogeneity, indicating that depression risk is shaped by both shared and context-specific influences.

**Conclusions:**

By integrating large-scale international cohort data with explainable machine learning, this study identified cross-national patterns of feature importance for concurrent depressive symptom status among middle-aged and older adults with chronic conditions. Poor self-rated health, somatic pain, and functional limitations may support cross-sectional screening and further assessment but should not be interpreted as early warning indicators.

## 1 Introduction

Driven by the global trajectory of population aging, mental health in middle-aged and older adults has transcended individual clinical concerns to become a pivotal issue linked to healthy aging, the sustainability of social care systems, and the efficacy of public health governance (1, 2). Among various conditions in middle-aged and older adults, depressive symptoms carry substantial public health implications. Global epidemiological evidence underscores a heavy disease burden associated with late-life depression, attracting widespread clinical and academic attention (3, 4). These symptoms are not only highly prevalent but also serve as critical precursors to functional decline, cognitive impairment, and increased mortality. Consequently, late-life depression represents more than a mere emotional disturbance; it functions as a sentinel indicator of adverse health outcomes (5–7). Unlike depression in younger cohorts, Depression in middle-aged and older adults is rarely the product of isolated psychosocial stressors. Instead, it typically emerges from a complex, chronic interplay of physiological degeneration, somatic burden, functional limitations, and environmental stressors (8, 9). Accordingly, its development is best conceptualized not as a simple summation of independent risk factors, but as a progressive accumulation of multidimensional vulnerabilities (10–12). Importantly, this cumulative process is sensitive to national and societal contexts. While traditional drivers—such as socioeconomic status and loneliness—consistently influence depression, emerging contextual factors, including digital exclusion and the built environment, further reshape these risks (13–15). Given the substantial differences in healthcare systems and cultural expressions of distress, risk signals may exhibit both cross-cultural commonality and context-specific heterogeneity (16, 17). Therefore, a cross-national perspective is essential to identify cross-nationally stable predictive correlates while clarifying how risk patterns diverge across social environments (18, 19).

Current literature has accumulated extensive evidence on correlates of geriatric depression; however, the primary limitation lies not in a lack of data, but in the absence of an integrative analytical framework capable of explaining how multiple risk signals converge. Conventional studies often treat variables as discrete factors, focusing on linear associations rather than examining how these elements organize into structurally meaningful vulnerability profiles. As a result, the transition from general health decline to high-risk emotional states remains insufficiently characterized (20, 21). Furthermore, most existing evidence derives from single-country cohorts, reflecting localized patterns that may not generalize globally. It remains unclear which predictive correlates show consistent feature contributions across countries and institutional contexts (22, 23). Recent studies have also applied interpretable machine learning to depression prediction. Chike et al. used BRFSS data and an XGBoost–SHAP framework to identify behavioral and socioeconomic predictors of depression, whereas Wang et al. developed an XGBoost–SHAP model for geriatric depression screening using NHANES data (38, 39). However, both studies were based on single-country datasets, and the cross-national stability of predictive signals remains unclear.

To address these gaps, the research priority must shift from merely cataloging variables to developing an analytical framework that captures complex relationships while maintaining high interpretability. While traditional statistical methods excel at estimating average effects, they often struggle to map the nonlinear interactions and hierarchical structures inherent in depression risk. Recent advances suggest that Machine Learning (ML) offers superior multivariable modeling capacity, while Explainable Artificial Intelligence (XAI) approaches bridge the gap between predictive power and clinical transparency (24, 25). For a multidimensional outcome like late-life depression, XAI serves as more than a computational tool; it provides an analytical pathway for characterizing feature-contribution patterns (26, 27). By integrating cross-national designs with an XAI framework, this study aims to move beyond a simple “list of correlates” toward the identification of cross-nationally stable and context-specific predictive correlates, providing a more robust evidence base for cross-sectional screening and further assessment in aging populations.

## 2 Methods

### 2.1 Study participants and overall process

In this population-based cross-national analysis, cross-sectional data were drawn from six aging cohorts comprising middle-aged and older adults aged 50 years and above across 36 countries: the China Health and Retirement Longitudinal Study (CHARLS, Wave 4), the English Longitudinal Study of Ageing (ELSA, Wave 9), the Health and Retirement Study (HRS, Wave 14), the Korean Longitudinal Study of Aging (KLoSA, Wave 6), the Mexican Health and Aging Study (MHAS, Wave 4), and the Survey of Health, Ageing and Retirement in Europe (SHARE, Wave 8). The study population was restricted to participants aged 50 years or older who reported at least one chronic condition. This restriction was adopted because the study was specifically designed to characterize depressive-symptom vulnerability in a clinically relevant population experiencing ongoing chronic health burden, rather than in the general aging population. Accordingly, the inclusion criterion was intended to define the target population consistently across cohorts, not to generate estimates generalizable to all middle-aged and older adults. The following exclusion criteria were applied: (1) missing data on depressive symptom variables; (2) missing data on individual-level variables; and (3) reporting no chronic condition diagnosed by a physician or other health professional in the corresponding survey wave.

**Figure 1:**
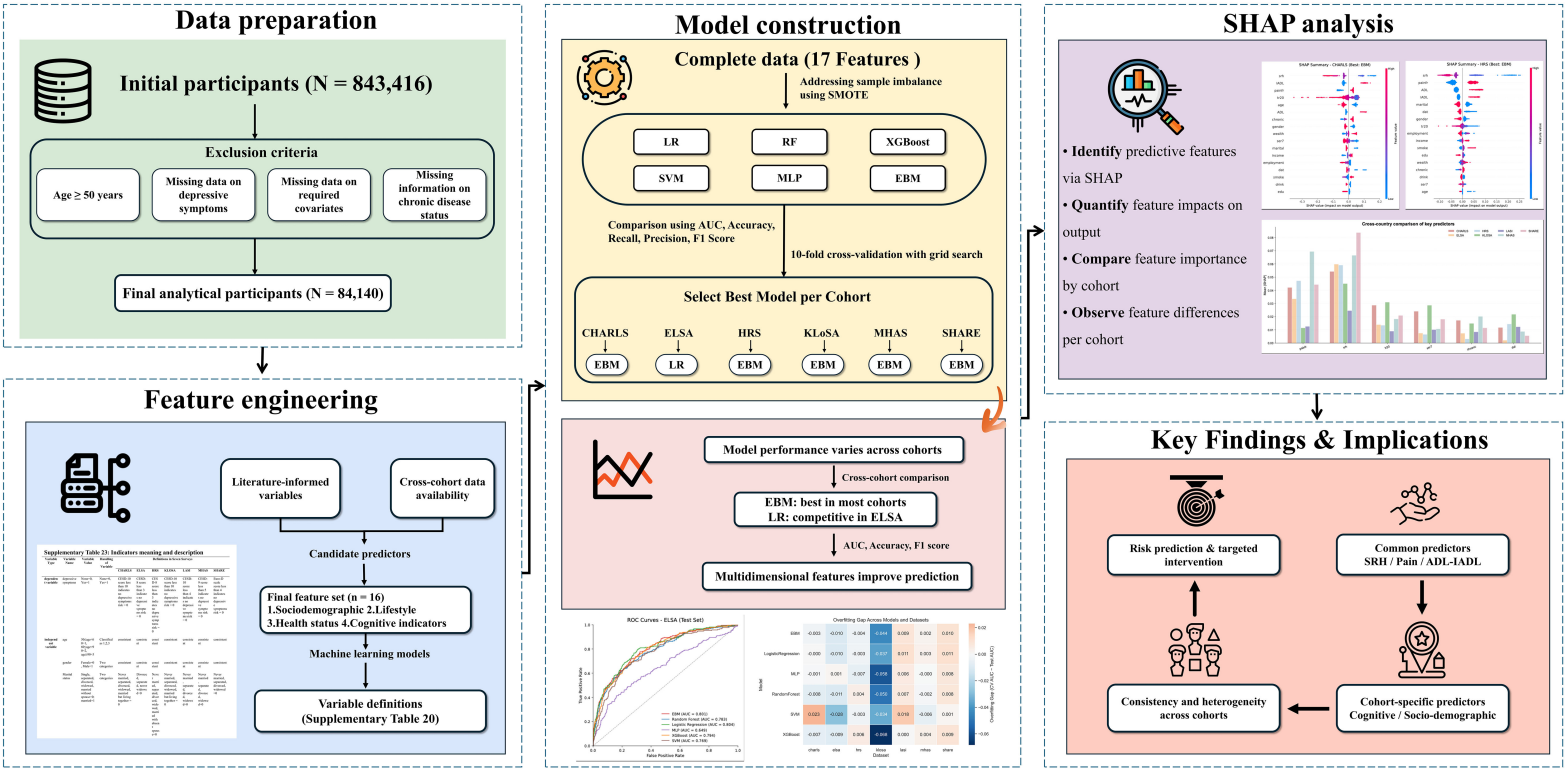
Integrated workflow for multi-cohort harmonization and interpretable machine learning analysis.

Chronic diseases were defined based on participants’ self-reports that a physician or other health professional had diagnosed the condition; these diagnoses were not independently verified using medical records, including stroke, hypertension, diabetes, chronic lung disease, heart disease, dyslipidemia, cancer, liver disease, kidney disease, gastric disease, psychiatric disorder, arthritis, rheumatic disease, respiratory disease, memory-related disorder, Parkinson’s disease, and cataract. After sample screening, the final analytic sample comprised 84,140 participants: 6,734 from CHARLS, 6,158 from ELSA, 7,761 from HRS, 6,704 from KLoSA, 13,007 from MHAS, and 43,776 from SHARE. This harmonization strategy was intended to enhance the representativeness of middle-aged and older populations across diverse cultural and socioeconomic settings and to support the systematic investigation of determinants of depressive symptom risk. A cross-national integrated explainable machine learning framework was then established, and the overall study design and analytical workflow are illustrated in **Figure 1**.

The workflow integrated the full process from data preparation and multidimensional feature engineering to model development and performance evaluation. First, the six global aging cohorts (N = 84,140) were harmonized and rigorously screened, resulting in a 17-feature system encompassing sociodemographic characteristics, lifestyle factors, health status, and cognitive function. Subsequently, six machine learning algorithms, including logistic regression, random forest, and explainable boosting machine (EBM), were used to develop prediction models, and synthetic minority over-sampling technique (SMOTE) was introduced to balance class distribution. Finally, SHAP analysis was applied to quantify feature contributions and to characterize cross-nationally stable and context-specific feature-contribution patterns, thereby providing an empirical basis for precision-oriented intervention in depressive symptoms among middle-aged and older adults.

### 2.2 Variables and definitions

On the basis of previous literature (28–31) and variable availability across multiple cohorts, a candidate variable system was constructed from four dimensions: sociodemographic background, lifestyle, health status, and subjective symptom–cognitive burden. This framework was adopted because depressive symptoms in middle-aged and older adults are unlikely to be driven by a single exposure; rather, they are more plausibly shaped by the combined influence of social structural factors, behavioral factors, physical health impairment, functional decline, and cognitive vulnerability.

Specifically, demographic and socioeconomic characteristics included age (agey), gender(ragender), marital status (mstath), educational attainment (raeducl), total household wealth (atotb_quartile), total household income (itot_quartile), and employment status (work), which were used to characterize participants’ basic social position and resource conditions. Lifestyle factors included smoking (smoke) and alcohol drinking (drink). Health-related indicators included self-rated health (SRH), activities of daily living (ADL), instrumental activities of daily living (IADL), and chronic disease burden (chronic), which were used to reflect overall health status, functional status, and accumulated physical disease burden. Subjective symptom and cognitive indicators included pain burden (painfr), word memory (trX), date orientation (dat), and executive function (ser7), which were incorporated to capture symptom experience and cognitive vulnerability. By including these variables, potential determinants of depressive symptom risk could be examined from multidimensional socioeconomic, behavioral, health-related, and cognitive perspectives.

A binary outcome variable was used to define depressive symptom risk as either present (1) or absent (0). Cohort-specific thresholds were defined as follows: CESD-8 ≥3 for HRS and ELSA, CESD-10 ≥10 for CHARLS and KLoSA, CESD-9 ≥5 for MHAS, and EURO-D ≥4 for SHARE. Scores below the corresponding thresholds were classified as indicating no depressive symptom risk. Evidence from previous literature (32) on the correlation between EURO-D and CESD supported the comparability of these measures in harmonized analyses, thereby ensuring consistency in the binary outcome definition despite differences in scale format.

To ensure comparability of key variables across the six cohorts, all variables were harmonized (**Supplementary Table 20**). In accordance with previous studies on depression threshold setting and variable harmonization in multinational cohorts (33), variables were recoded according to unified principles because the original scale formats and scoring systems differed across surveys. Specifically, age 1 = 50–59 years, 2 = 60–89 years, and 3 = ≥90 years; gender 0 = female and 1 = male; marital status 0 = unmarried/other and 1 = married; education 1 = junior high school or below, 2 = high school, and 3 = higher education; wealth and income range from 1 (highest quartile) to 4 (lowest quartile); employment 1 = unemployed/not working, 2 = retired, and 3 = working; smoking 1 = never, 2 = former, and 3 = current; drinking 0 = no and 1 = yes; self-rated health ranges from 1 (very poor) to 5 (very good/excellent); ADL/IADL 0 = no difficulty and 1 = any difficulty; chronic disease count 1 = one, 2 = two, and 3 = three or more conditions; pain 0 = absent and 1 = present.

### 2.3 Data preprocessing

**Figure 2:**
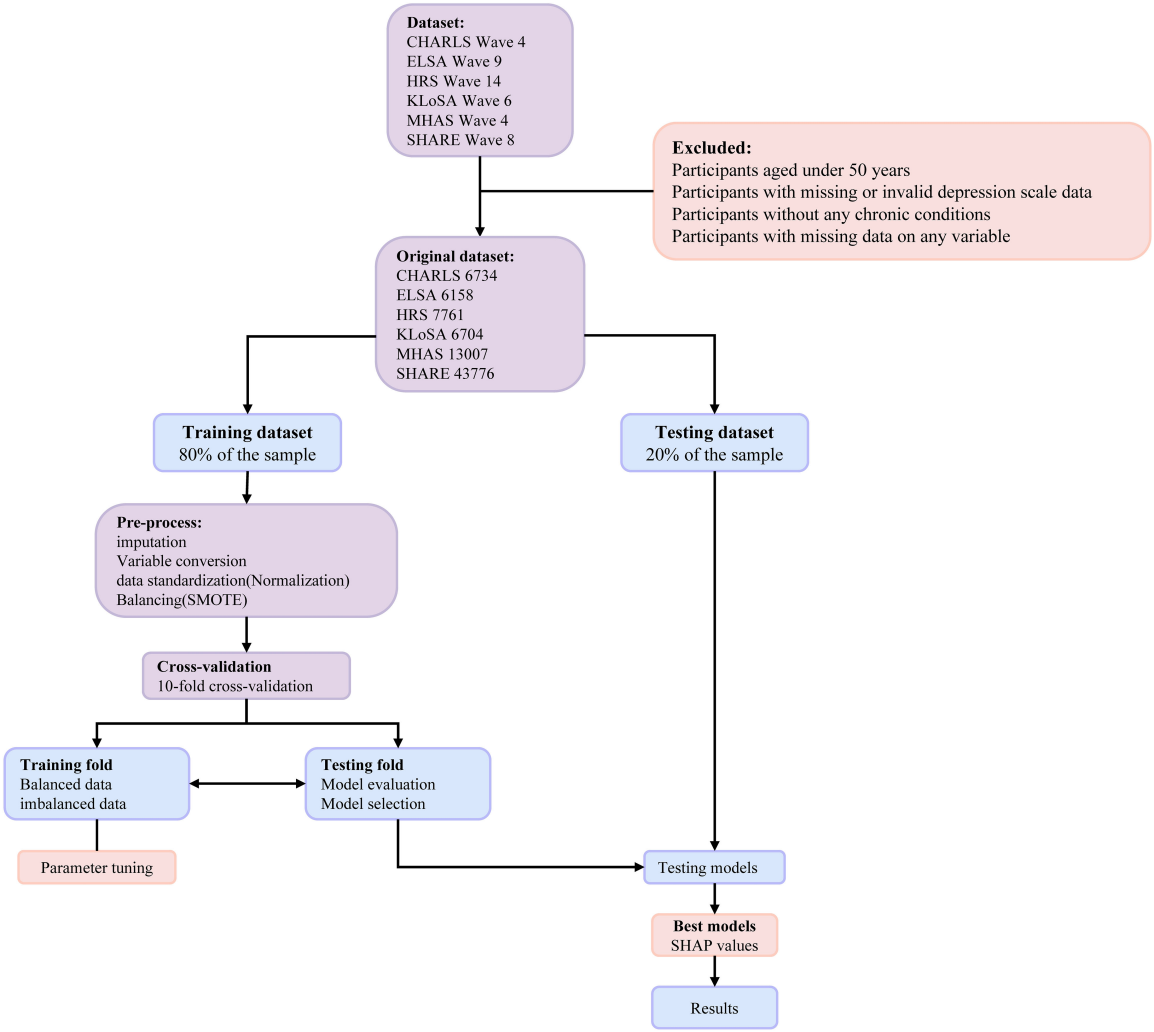
Flowchart of the integrated predictive research across cohorts.

The workflow for data preprocessing and model development is shown in **Figure 2**. Feature engineering was conducted before model training in order to transform raw data into a form more suitable for modeling, thereby improving model stability and generalizability. This process mainly consisted of data cleaning, feature selection, and feature transformation. First, the raw data were organized and screened to remove redundant or irrelevant variables, thereby reducing noise and lowering the risk of overfitting. Variable selection was guided by prior literature and the data structures of the six public aging cohorts in order to improve cross-cohort comparability. At the same time, variable definitions were harmonized as far as possible while preserving necessary differences across survey systems.

The final 17 predictors were selected *a priori* before model development based on previous literature, availability across all six cohorts, and harmonizability across survey systems. Variables were excluded from the candidate set if they were unavailable in one or more cohorts, could not be consistently harmonized, or showed zero variance after harmonization. No univariate outcome-based screening or statistical significance threshold was used for predictor selection. Therefore, the final feature set was designed to support cross-cohort comparability rather than to maximize cohort-specific statistical associations. After the train–test split, all data-dependent preprocessing procedures were performed using the training data only to avoid information leakage (34).

Missingness was assessed separately for each cohort and variable before model development. After cohort-specific screening and harmonization, missing values in the final predictor set were handled within the model-development pipeline using SimpleImputer. To avoid information leakage, the imputation procedure was fitted using the training data only and then applied to the held-out test set. Continuous and ordinal variables were imputed using the median value from the training set, whereas categorical variables were imputed using the most frequent category from the training set (35). The imputed data were then used for model training and test-set evaluation. SMOTE was selected as the primary class-balancing strategy because it is a model-agnostic oversampling method that can be applied consistently across different algorithms and cohorts (36). Unlike class weighting or cost-sensitive learning, which may require model-specific implementation and tuning, SMOTE provided a uniform preprocessing strategy for the six prespecified algorithms. To avoid information leakage, SMOTE was applied only to the training data after the train–test split and was not applied to the held-out test set. Alternative imbalance-handling strategies, including ADASYN, Borderline-SMOTE, class weighting, and cost-sensitive learning, were not systematically benchmarked in the present study. Therefore, robustness analyses using the original imbalanced training data were also conducted and are reported in **Supplementary Tables 14**-**19**.

### 2.4 Predictive model development and evaluation

For each cohort, the dataset was randomly divided into a training set (80%) and a test set (20%). Six machine learning algorithms—XGBoost, logistic regression, random forest, support vector machine (SVM), multilayer perceptron (MLP), and explainable boosting machine (EBM)—were used to develop prediction models so that predictive performance could be comprehensively evaluated.

The adopted framework integrated six complementary machine learning methods. XGBoost represented an optimized gradient boosting implementation; logistic regression served as a classical probabilistic model; random forest represented an ensemble of decision trees; SVM was used to define an optimal decision boundary; MLP represented a feedforward artificial neural network capable of capturing nonlinear relationships; and EBM represented an interpretable algorithm based on generalized additive models. To further clarify determinants of depressive symptom risk, SHAP analysis was subsequently applied to the best-performing model in each cohort in order to interpret and visualize feature contributions.

Key hyperparameters for each model were optimized using grid search in combination with 10-fold cross-validation, with the area under the receiver operating characteristic curve (AUC) used as the primary tuning metric. AUC was selected because it provides an overall assessment of a model’s ability to discriminate between positive and negative cases across different classification thresholds, particularly in imbalanced binary classification tasks. Final model performance was then evaluated independently in the test set using AUC, accuracy, recall, precision, and F1-score. For each model and cohort, 95% confidence intervals for AUC, accuracy, precision, recall, and F1-score were estimated using stratified non-parametric bootstrap resampling of the held-out test set. Positive and negative cases were resampled separately at the participant level to preserve the outcome distribution. To assess whether observed differences between models were statistically meaningful, paired stratified bootstrap comparisons were further conducted for AUC differences. Within each cohort, the same bootstrap resampling indices were applied to all models, and Holm correction was used to adjust for multiple comparisons.

This prespecified model set enabled transparent and consistent cross-cohort comparisons using the same tuning and evaluation procedures. Automated benchmarking frameworks such as PyCaret were not adopted because the objective of the present study was a controlled comparison of complementary model families rather than an exhaustive search across all available algorithms. Nevertheless, such frameworks could be considered in future studies to evaluate a broader range of candidate models (37).

To further interpret model predictions and identify important risk factors, SHAP (SHapley Additive exPlanations) analysis was conducted for the best-performing model in each cohort. This method quantifies the contribution of each feature to model output and displays both its importance and direction of effect. Because SHAP values may be affected by correlations among predictors, we interpreted SHAP-based feature rankings as model-based attribution patterns rather than independent causal effects. Therefore, the SHAP results were used to compare relative feature contributions within each fitted model, while potential multicollinearity among predictors was evaluated descriptively using correlation matrices. Hyperparameter settings for the optimal models are provided in **Supplementary Table 21**, and the flowcharts of cross-sectional sample selection are shown in **Supplementary Figures 1**-**6**.

## 3 Results

### 3.1 Descriptive statistical analysis of variables

Descriptive statistical analyses showed that depressive symptoms were common in the overall sample and were distributed unevenly across multiple dimensions, including demographic characteristics, socioeconomic conditions, health status, and cognitive function. A total of 84,140 participants were included, of whom 21,960 (26.10%) were classified into the depressive symptom group and 62,180 (73.90%) into the non-depressive symptom group. With respect to age composition, participants aged 50–59 years, 60–89 years, and ≥90 years accounted for 20.34%, 77.79%, and 1.87% of the sample, respectively. The overall results are summarized in **Table 1**, with detailed cohort-specific results provided in **Supplementary Tables 1**-**7**.

**Table 1:** Baseline characteristics by depressive symptom status.

Characteristic	Overall	Depression	Non-depression	P	Characteristic	Overall	Depression	Non-depression	P
**Age group**				**<0.001**	**ADL limitation**				**<0.001**
1	17,117 (20.34)	4,318 (19.66)	12,799 (20.58)		0	73,100 (86.88)	16,030 (73.00)	57,070 (91.78)	
2	65,449 (77.79)	17,022 (77.51)	48,427 (77.88)		1	11,040 (13.12)	5,930 (27.00)	5,110 (8.22)	
3	1,574 (1.87)	620 (2.82)	954 (1.53)		**IADL limitation**				**<0.001**
**Gender**				**<0.001**	0	70,020 (83.22)	14,620 (66.58)	55,400 (89.10)	
0	47,190 (56.09)	14,534 (66.18)	32,656 (52.52)		1	14,120 (16.78)	7,340 (33.42)	6,780 (10.90)	
1	36,950 (43.91)	7,426 (33.82)	29,524 (47.48)		**Chronic disease count**				**<0.001**
**Marital status**				**<0.001**	1	18,523 (22.01)	2,859 (13.02)	15,664 (25.19)	
0	28,916 (34.37)	9,345 (42.55)	19,571 (31.47)		2	23,331 (27.73)	5,022 (22.87)	18,309 (29.45)	
1	55,224 (65.63)	12,615 (57.45)	42,609 (68.53)		3	42,286 (50.26)	14,079 (64.11)	28,207 (45.36)	
**Education level**				**<0.001**	**Pain distress**				**<0.001**
1	37,344 (44.38)	12,322 (56.11)	25,022 (40.24)		0	45,978 (54.64)	7,514 (34.22)	38,464 (61.86)	
2	30,407 (36.14)	6,703 (30.52)	23,704 (38.12)		1	38,162 (45.36)	14,446 (65.78)	23,716 (38.14)	
3	16,389 (19.48)	2,935 (13.37)	13,454 (21.64)		**Verbal memory**				**<0.001**
**Household wealth**				**<0.001**	≤ -2.5	385 (0.46)	179 (0.82)	206 (0.33)	
1	21,020 (24.98)	4,078 (18.57)	16,942 (27.25)		(-2.5, -2]	1,367 (1.62)	635 (2.89)	732 (1.18)	
2	21,045 (25.01)	4,857 (22.12)	16,188 (26.03)		(-2, -1.5]	4,302 (5.11)	1,749 (7.96)	2,553 (4.11)	
3	20,809 (24.73)	5,706 (25.98)	15,103 (24.29)		(-1.5, -1]	7,390 (8.78)	2,699 (12.29)	4,691 (7.54)	
4	21,266 (25.27)	7,319 (33.33)	13,947 (22.43)		(-1, -0.5]	11,779 (14.00)	3,653 (16.63)	8,126 (13.07)	
**Household income**				**<0.001**	(-0.5, 0]	15,715 (18.68)	4,174 (19.01)	11,541 (18.56)	
1	20,952 (24.90)	3,866 (17.60)	17,086 (27.48)		(0, 0.5]	13,670 (16.25)	3,138 (14.29)	10,532 (16.94)	
2	21,025 (24.99)	4,895 (22.29)	16,130 (25.94)		(0.5, 1]	16,447 (19.55)	3,227 (14.69)	13,220 (21.26)	
3	21,023 (24.99)	5,774 (26.29)	15,249 (24.52)		(1, 1.5]	8,298 (9.86)	1,664 (7.58)	6,634 (10.67)	
4	21,140 (25.12)	7,425 (33.81)	13,715 (22.06)		(1.5, 2]	3,470 (4.12)	643 (2.93)	2,827 (4.55)	
**Employment status**				**<0.001**	(2, 2.5]	1,055 (1.25)	162 (0.74)	893 (1.44)	
1	24,861 (29.55)	5,274 (24.02)	19,587 (31.50)		> 2.5	262 (0.31)	37 (0.17)	225 (0.36)	
2	14,765 (17.55)	5,671 (25.82)	9,094 (14.63)		**Orientation to date**				**<0.001**
3	44,514 (52.90)	11,015 (50.16)	33,499 (53.87)		0	1,386 (1.65)	641 (2.92)	745 (1.20)	
**Smoking**				**<0.001**	1	2,530 (3.01)	1,111 (5.06)	1,419 (2.28)	
1	47,263 (56.17)	12,684 (57.76)	34,579 (55.61)		2	12,114 (14.40)	4,105 (18.69)	8,009 (12.88)	
2	24,770 (29.44)	5,928 (26.99)	18,842 (30.30)		3	68,110 (80.95)	16,103 (73.33)	52,007 (83.64)	
3	12,107 (14.39)	3,348 (15.25)	8,759 (14.09)		**Executive function**				**<0.001**
**Drinking**				**<0.001**	0	2,930 (3.48)	1,305 (5.94)	1,625 (2.61)	
0	45,177 (53.69)	14,135 (64.37)	31,042 (49.92)		1	6,862 (8.16)	2,690 (12.25)	4,172 (6.71)	
1	38,963 (46.31)	7,825 (35.63)	31,138 (50.08)		2	6,274 (7.46)	2,213 (10.08)	4,061 (6.53)	
**Self-rated health**				**<0.001**	3	10,737 (12.76)	3,180 (14.48)	7,557 (12.15)	
1	7,316 (8.70)	4,307 (19.61)	3,009 (4.84)		4	14,621 (17.38)	3,886 (17.70)	10,735 (17.26)	
2	19,573 (23.26)	6,727 (30.63)	12,846 (20.66)		5	42,716 (50.77)	8,686 (39.55)	34,030 (54.73)	
3	30,252 (35.95)	5,719 (26.04)	24,533 (39.45)						
4	19,970 (23.73)	3,528 (16.07)	16,442 (26.44)						
5	7,029 (8.35)	1,679 (7.65)	5,350 (8.60)						

Values are presented as n (%). Overall n = 84,140; depression n = 21,960; non-depression n = 62,180.

Data are presented as n (%). Between-group differences were assessed using two-sided Pearson chi-square tests. P values represent comparisons between participants with and without depressive symptoms.

Bold P-values indicate statistical significance (P < 0.05).

More pronounced vulnerability patterns were observed in the depressive symptom group with regard to demographic and socioeconomic characteristics. The proportion of women was higher in the depressive symptom group than in the non-depressive symptom group (66.18% vs 52.52%), as was the proportion of unmarried individuals (42.55% vs 31.47%). Low educational attainment was also more common in the depressive symptom group (56.11% vs 40.24%). Significant between-group differences were additionally observed in household wealth, household income, and employment status, with the depressive symptom group being more concentrated in lower socioeconomic strata; the proportion of retired individuals was also higher in this group (25.82% vs 14.63%; all P<0.001).

Health-related indicators exhibited the most prominent between-group differences. Poorer SRH was markedly enriched in the depressive symptom group, with the combined proportion reporting “poor” or “very poor” health reaching 50.24%, compared with 25.50% in the non-depressive symptom group. Difficulties in ADL and IADL were reported by 27.00% and 33.42% of the depressive symptom group, respectively, compared with 8.22% and 10.90% in the non-depressive symptom group. Likewise, the proportion of individuals with ≥3 chronic conditions was higher in the depressive symptom group than in the non-depressive symptom group (64.11% vs 45.36%). Pain burden was also more common in the depressive symptom group (65.78% vs 38.14%; all P<0.001).

Consistent differences were also observed in cognitive function. The proportion of participants with the highest level of temporal orientation (score = 3) was lower in the depressive symptom group than in the non-depressive symptom group (73.33% vs 83.64%), and the proportion achieving the highest executive function score (score = 5) was likewise lower (39.55% vs 54.73%). The distribution of word memory further indicated that the depressive symptom group was more concentrated in lower memory ranges, whereas the non-depressive symptom group was more frequently represented in higher memory ranges (all P<0.001). Overall, depressive symptoms were associated with clear distributional differences in demographic vulnerability, socioeconomic disadvantage, deteriorated health status, functional limitation, pain burden, and cognitive decline. Among these, health deterioration, functional impairment, multimorbidity, and pain burden emerged as the most prominent co-occurring characteristics in the depressive symptom group, thereby providing the basis for subsequent multivariable analyses.

### 3.2 Evaluation of model performance

**Figure 3:**
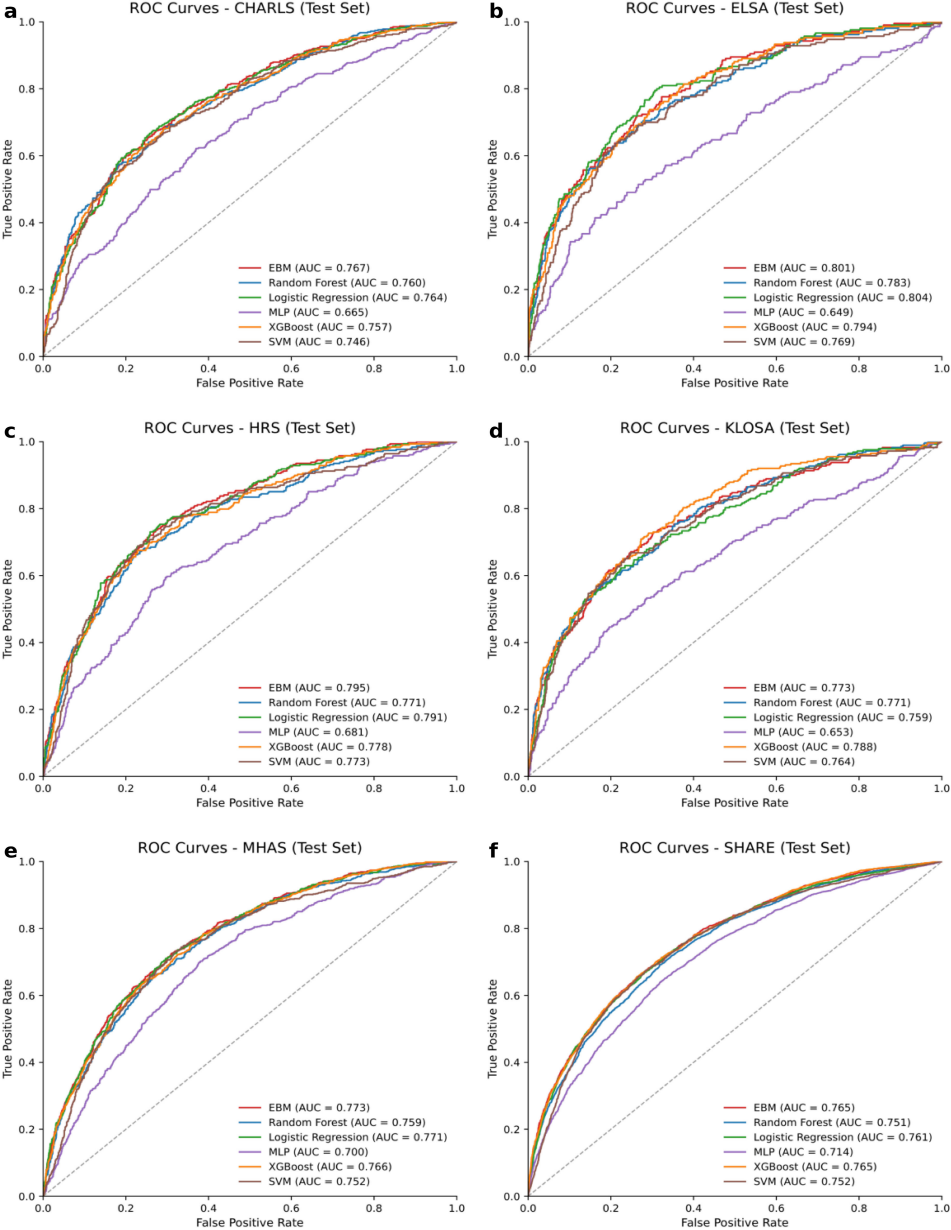
ROC curves for machine learning models predicting the risk of depressive symptoms in older adults across six cohorts: **(A)** CHARLS, **(B)** ELSA, **(C)** HRS, **(D)** KLoSA, **(E)** MHAS, and **(F)** SHARE.

The predictive performance of six machine learning models—LR, RF, SVM, MLP, XGBoost, and EBM—was systematically evaluated across six international longitudinal aging cohorts: CHARLS, ELSA, HRS, KLoSA, MHAS, and SHARE. The models achieved moderate predictive performance across cohorts, with AUC values ranging from 0.77 to 0.80. The results are shown in **Figure 3**, and the AUC, accuracy, precision, recall, and F1-score, together with their 95% confidence intervals, are reported in **Supplementary Tables 8**-**13**. Given the relatively low proportion of participants with depressive symptoms, SMOTE was applied to the training data for class balancing. Robustness analyses were further conducted using the original imbalanced training data, and the corresponding performance estimates with 95% confidence intervals are presented in **Supplementary Tables 14**-**19**. These analyses were used to examine whether model performance patterns were sensitive to oversampling. Because alternative imbalance-handling strategies were not systematically benchmarked, the SMOTE-based results should be interpreted as one prespecified class-balancing approach rather than as evidence that SMOTE is superior to other methods.

Overall, EBM achieved the highest or near-highest AUC in most cohorts and was the most stable-performing model, although its advantage was generally limited to marginal improvement. In CHARLS, KLoSA, MHAS, and SHARE, the AUCs achieved by EBM were 0.7659, 0.7762, 0.7734, and 0.7653, respectively, representing the best performance in each of these cohorts. In HRS, the AUC of EBM was 0.7941, only slightly higher than that of LR (0.7915). In ELSA, LR and EBM performed almost identically, with AUCs of 0.8032 and 0.8029, respectively. Across cohorts, the best-performing models yielded AUCs ranging from 0.7653 to 0.8032. Performance was relatively better in ELSA and HRS, whereas CHARLS, KLoSA, MHAS, and SHARE showed broadly comparable performance levels, suggesting that the current variable system provided a certain degree of discriminative capacity across populations from different countries. EBM and logistic regression performed nearly identically in most cohorts, with absolute AUC differences below 0.005 in five of six cohorts.

Although the overall AUC values fell within an acceptable range, recall results suggested that the ability of the models to identify individuals with depressive symptoms remained limited. In general, the models were able to distinguish the overall sample to a certain extent, but detection of the minority class was still insufficient, indicating a shared limitation of the current prediction framework: acceptable overall discrimination but inadequate sensitivity.

### 3.3 SHAP analysis of feature contributions

**Figure 4:**
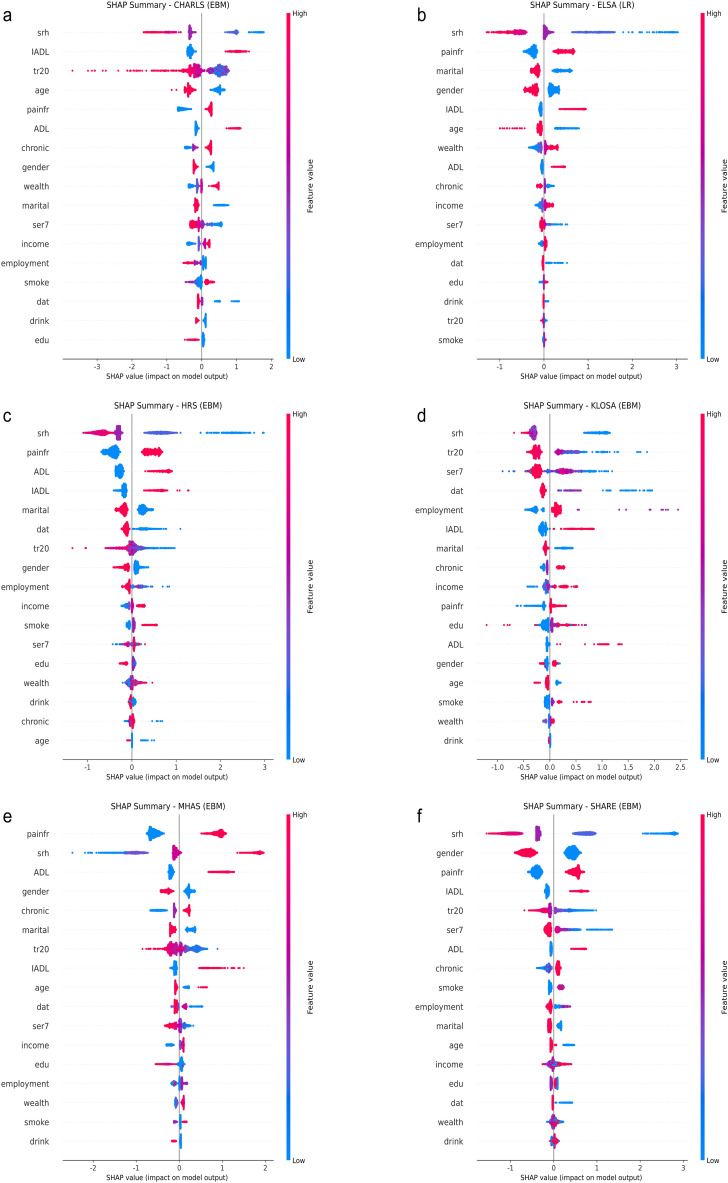
SHAP summary plots for the optimal machine learning models across six cohorts: **(A)** CHARLS, **(B)** ELSA, **(C)** HRS, **(D)** KLoSA, **(E)** MHAS, and **(F)** SHARE.

SHAP was used to interpret the feature contributions of the best-performing model in each cohort and to compare the relative importance of different variables in predicting depressive symptoms among middle-aged and older adults. As shown in **Figure 4**, the feature-contribution patterns observed across the six national cohorts exhibited both stable cross-cohort commonality and marked contextual heterogeneity. SRH emerged as the most stable core predictor, ranking first in CHARLS, ELSA, HRS, KLoSA, and SHARE, and second in MHAS, thereby indicating that it was the most consistent high-contribution signal across cultural contexts.

Pain burden and functional limitation constituted a second relatively stable predictive dimension. Pain burden ranked among the leading features in ELSA, HRS, MHAS, and SHARE, and exceeded SRH in MHAS, where it became the highest-contributing feature. At the same time, IADL and ADL remained highly ranked across cohorts, with IADL being more prominent in CHARLS, whereas ADL contributed more strongly in HRS and MHAS. These findings suggest that bodily discomfort and loss of functional independence represent relatively consistent structural dimensions in the prediction of depressive symptoms.

By contrast, cognitive, socioeconomic, and demographic variables exhibited stronger cohort specificity. TrX and dat ranked highly in KLoSA, indicating that cognitive indicators played a more prominent role in the Korean cohort. Household wealth and household income entered the leading ranks in CHARLS, suggesting a stronger contribution of socioeconomic factors in the Chinese cohort. In ELSA and SHARE, gender and marital status were relatively more important, reflecting a stronger explanatory role of social relations and demographic characteristics in some European cohorts. Overall, these variables did not show the same degree of cross-cohort consistency as SRH, pain burden, and functional status, but instead appeared to reflect more context-dependent heterogeneity.

Taken together, the SHAP results suggest that the predictive pattern of depressive symptoms in middle-aged and older adults is not driven by a single factor, but instead reflects a multidimensional structure in which shared core signals coexist with cohort-specific modifying factors. SRH, pain burden, and functional limitation constituted the most stable core dimensions across cohorts, whereas cognitive, socioeconomic, and demographic variables appeared to shape the specific expression of risk in different countries. It should be noted that SHAP reflects the relative contribution of variables to model output rather than independent causal effects; accordingly, these findings are more appropriate for cross-cohort comparison and risk stratification than for causal interpretation.

### 3.4 Cross-cohort distribution of feature importance

**Figure 5:**
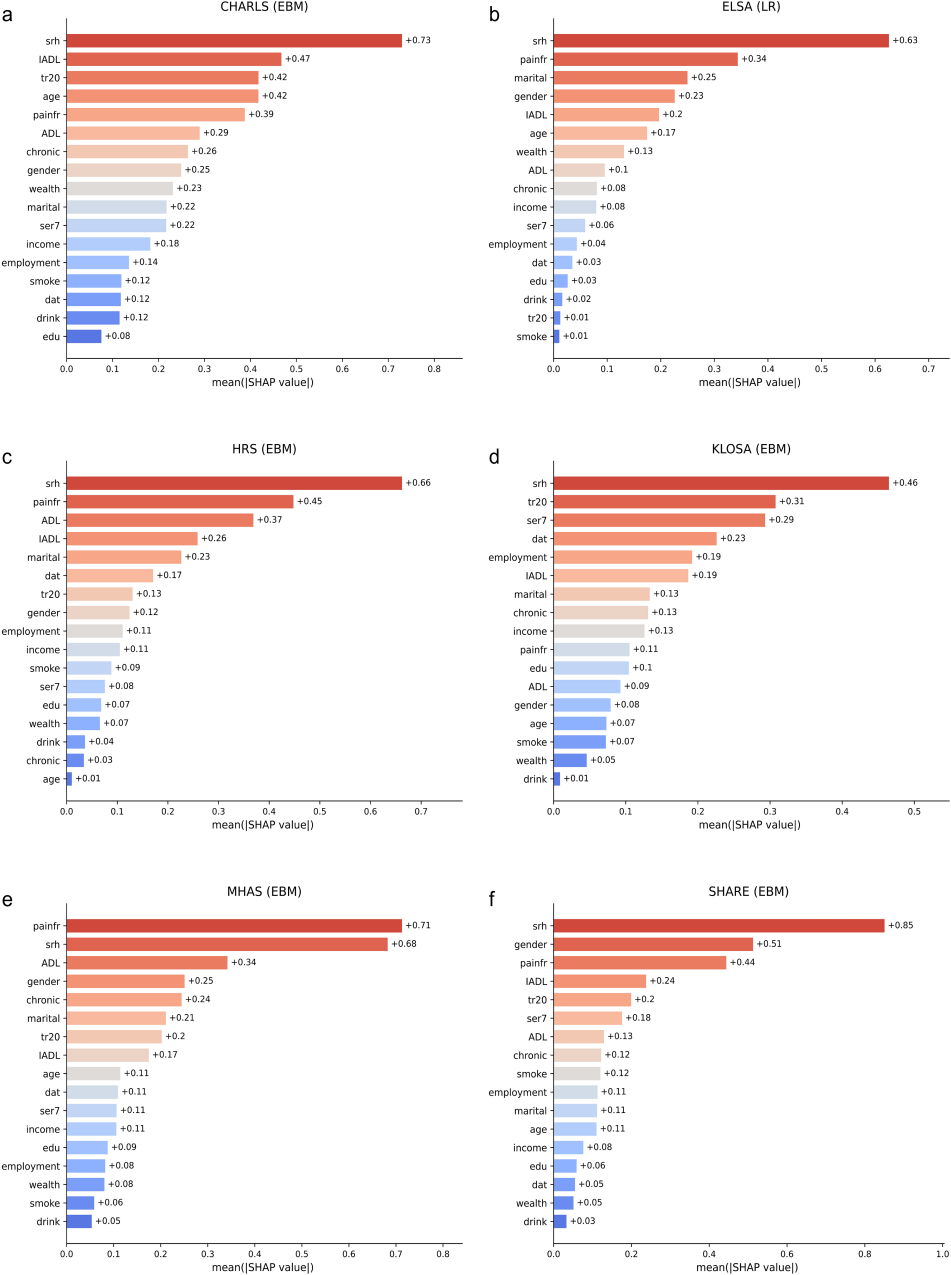
Feature importance ranking for the optimal machine learning models across six cohorts: **(A)** CHARLS, **(B)** ELSA, **(C)** HRS, **(D)** KLoSA, **(E)** MHAS, and **(F)** SHARE.

**Figure 5** illustrates the distribution of SHAP-derived feature contributions across the six national cohorts. Overall, concurrent depressive symptom status in middle-aged and older adults displayed both cross-cohort commonality and pronounced heterogeneity. SRH was the most stable core feature, ranking first in CHARLS, ELSA, HRS, KLoSA, and SHARE, and second in MHAS.

In addition to SRH, pain burden and functional limitation represented another relatively stable set of important features. Pain burden ranked among the leading features in CHARLS, ELSA, HRS, MHAS, and SHARE, and was ranked first in MHAS. IADL and ADL also maintained relatively high contributions across cohorts, although their relative importance differed: IADL was more prominent in CHARLS and SHARE, whereas ADL ranked higher in HRS and MHAS. These findings indicate that bodily discomfort and reduced independence in daily life represent relatively consistent dimensions of risk.

In contrast, cognitive, demographic, and socioeconomic features exhibited stronger cohort specificity. Cognitive-related features were most prominent in KLoSA, where word memory, executive function, and date orientation all ranked among the leading predictors. Gender contributed more strongly in SHARE and MHAS, whereas marital status was more prominent in ELSA and HRS. By comparison, income, wealth, education, smoking, and alcohol use generally occupied middle or lower ranks. Overall, **Figure 5** indicates that concurrent depressive symptom status in middle-aged and older adults is shaped jointly by SRH, pain burden, functional limitation, cognitive status, and sociodemographic context, with SRH remaining the most stable cross-cohort core feature.

### 3.5 Cross-country stability and risk-stratified feature-contribution patterns

To further characterize the core structure of concurrent depressive symptom status in middle-aged and older adults, six core predictors were selected from the overall SHAP results on the basis of cross-cohort consistency and theoretical interpretability: SRH, painfr, trX, ser7, dat, and chronic.

Cross-country comparison showed that SRH was the most stable core signal, as illustrated in **Supplementary Figure 7**. SRH had the highest or near-highest mean absolute SHAP value in most cohorts, and its cross-cohort consistency was clearly stronger than that of the other variables. By comparison, painfr also maintained relatively high contributions across most cohorts, but showed greater between-cohort fluctuation, suggesting that although it was broadly important, it was also more context-dependent. trX, ser7, dat, and chronic showed higher contributions only in selected cohorts and were therefore more reflective of country-specific supplementary signals. Overall, **Supplementary Figure 7** revealed a relatively clear layered pattern: SRH constituted the most stable cross-national core layer, painfr represented a broadly important secondary core layer, and cognitive- and chronic disease-related variables were more characteristic of context-specific supplementary layers.

**Figure 6:**
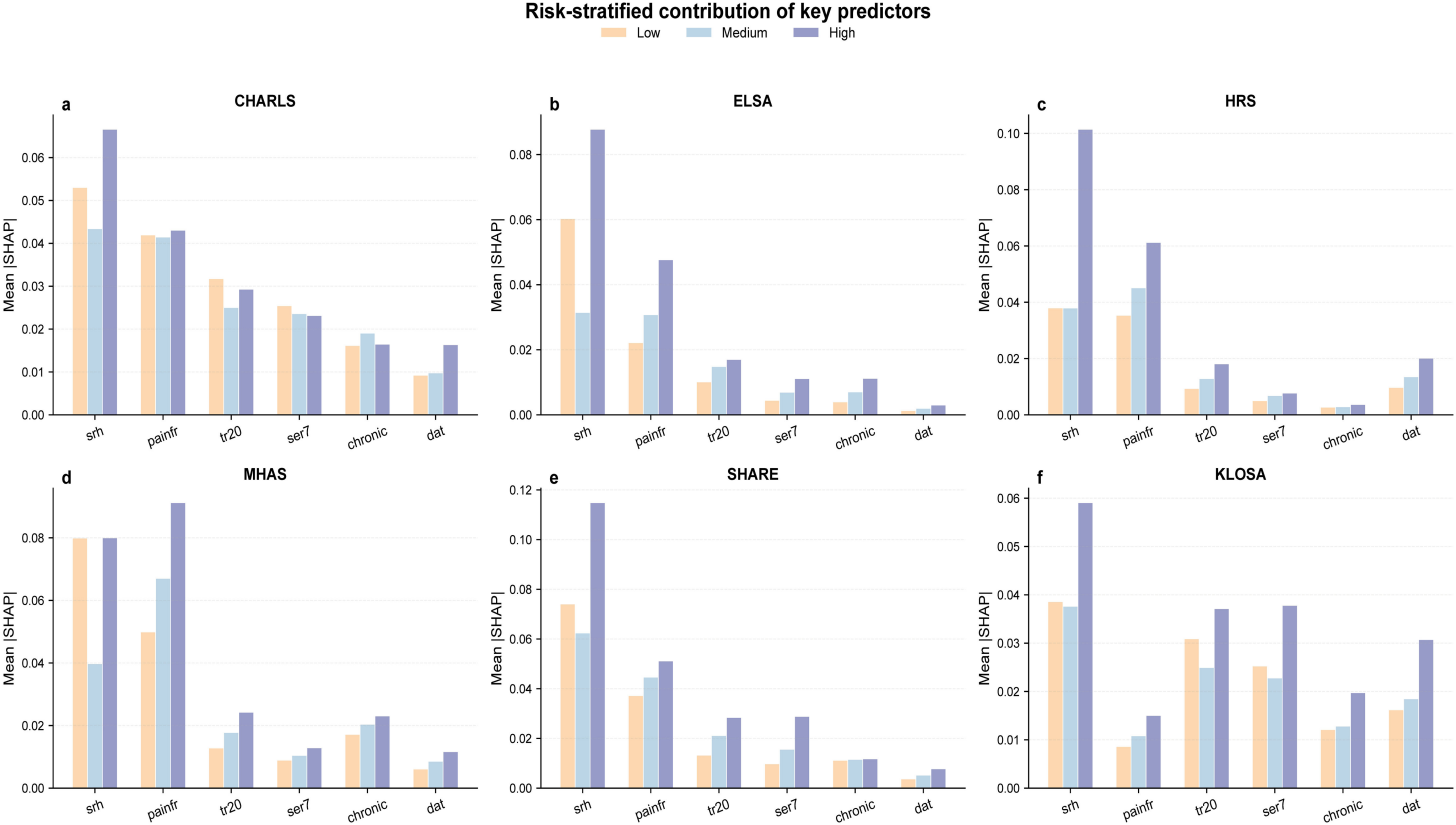
Comparison of key predictor contributions across risk strata in six cohorts: **(A)** CHARLS, **(B)** ELSA, **(C)** HRS, **(D)** MHAS, **(E)** SHARE, and **(F)** KLoSA.

Further risk-stratified analyses showed that the amplification patterns of the core variables were not uniform as risk increased; the results are shown in **Figure 6**. The contribution of SRH increased consistently from the low-risk to the high-risk strata in most cohorts, suggesting that it not only exhibited the strongest cross-national stability but also most closely approximated the principal axis of high-risk formation. painfr likewise tended to increase with rising risk in most cohorts, although the magnitude of this increase varied more substantially across countries. In contrast, trX, ser7, dat, and chronic showed increased contributions in high-risk groups only in selected cohorts, suggesting that these variables may function more as supplementary amplifiers under specific social or health-related contexts.

Overall, **Supplementary Figure 7**; **Figure 6** jointly indicate that depressive symptom risk in middle-aged and older adults is not best understood as the simple juxtaposition of multiple factors, but rather as a layered structure in which shared core signals coexist with context-specific amplifying signals. Among these, SRH most closely satisfies the dual criteria of cross-national stability and progressive amplification across risk strata, making it the signal most closely aligned with the principal axis of high-risk formation. painfr appeared secondary to SRH in this regard, whereas cognitive- and chronic disease-related variables functioned more as supplementary amplifying factors under specific contextual conditions.

## 4 Discussion

This study systematically characterized the cross-national structure of depressive symptom risk in middle-aged and older adults by integrating six international aging cohorts within an explainable machine learning framework. The results showed that, although a certain degree of heterogeneity existed across countries, the observed risk patterns were not best understood as fragmented local constellations of predictors, but rather as a relatively clear layered structure. Descriptive analyses indicated that individuals with depressive symptoms were more heavily concentrated among those with poorer health status, greater functional limitation, heavier pain burden, and worse cognitive performance. Subsequent model-based and SHAP analyses further suggested that these differences did not simply correspond to a set of parallel risk factors; instead, they could be organized within a framework in which stable core signals coexisted with context-specific signals. Compared with these single-country studies, the present study extends interpretable depression prediction by evaluating the reproducibility of vulnerability signals across six international aging cohorts (28, 29).

The most important finding was that SRH demonstrated the most stable and sustained predictive contribution across countries. Across feature rankings, cross-cohort comparisons, and risk-stratified analyses, SRH consistently remained among the most influential signals and became further amplified in higher-risk strata. Pain burden and functional limitation also retained substantial contributions in most cohorts, constituting a second relatively stable dimension of vulnerability. Taken together, these findings suggest that the common axis of depressive symptom risk in middle-aged and older adults is not primarily anchored in any single disease diagnosis or isolated socioeconomic indicator. Rather, it is more centrally expressed as an overarching process shaped by declining subjective health perception, accumulating bodily discomfort, and diminishing functional independence. Thus, what was identified in this study was not merely a static ranking of important variables, but a recurring logic of risk organization across countries.

However, the presence of this common axis should not be interpreted as evidence that national feature-contribution patterns are fully homogeneous. Relative to SRH, pain burden, and functional limitation, cognitive, socioeconomic, and demographic factors exhibited more pronounced cohort specificity. Cognitive indicators were more prominent in KLoSA, socioeconomic factors contributed more strongly in CHARLS, and gender and marital status carried greater explanatory weight in ELSA and SHARE. These differences suggest that depressive symptom risk in later life is underpinned not only by a shared cross-national vulnerability base, but also by continuous shaping through specific social contexts. Cross-national variation should therefore be understood not as a contradiction of the common pattern, but as an extension of its layered expression. The near-equivalence of LR and EBM in most cohorts indicates that complex algorithms provided little incremental discrimination. The limited performance gain may reflect the harmonized 17-feature framework and the possibility that much of the predictive information was captured by largely additive health-related variables. Accordingly, the primary contribution of the study lies in interpretable cross-cohort comparison rather than predictive superiority.

From a public health perspective, the findings suggest that risk identification in later life should balance stability with contextual sensitivity. The importance of SRH, pain burden, and functional limitation lies not only in their predictive strength, but also in their transferability across countries as baseline signals for early identification. By contrast, cognitive status, family structure, and socioeconomic position appear to function more as context-dependent modifiers whose weights vary across settings. Accordingly, a more interpretable and generalizable framework for identifying late-life depressive risk may be achieved by moving beyond horizontal comparisons of variable importance toward a layered understanding of shared core signals and contextual modifiers. For primary care screening and stratified prevention, such a perspective may be more informative than pursuing only marginal gains in model performance, because it better reflects how risk accumulates and how resources are allocated in practice.

Several limitations should be acknowledged. First, because the analyses were based on observational data, the SHAP results reflect the relative contribution of variables to model output rather than independent causal effects. Second, although SMOTE was used to address class imbalance, alternative strategies such as ADASYN, Borderline-SMOTE, class weighting, and cost-sensitive learning were not systematically compared. Different imbalance-handling strategies may influence the precision–recall trade-off, calibration, and feature-attribution patterns. Therefore, future studies should benchmark multiple balancing strategies within the same cross-validation framework to evaluate their effects on discrimination, minority-class detection, and interpretability. Third, restricting the analytic sample to participants with at least one chronic condition limits the generalizability of the findings to the broader middle-aged and older population and may increase the apparent contribution of health-related predictors, including self-rated health, pain burden, ADL, and IADL. Because a sensitivity analysis including participants without chronic conditions was not conducted, the stability of these findings in the general aging population remains uncertain. Future studies should include participants with and without chronic conditions to evaluate the robustness of the identified predictive patterns.

## 5 Conclusion

In conclusion, this study advances the understanding of mid- and late-life depressive symptoms from the listing of individual correlates to the identification of an interpretable cross-national feature-contribution patterns. By integrating six international aging cohorts within an explainable machine learning framework, we show that depressive risk in middle-aged and older adults with chronic conditions is better understood as a layered structure, in which a limited set of cross-culturally stable core signals is embedded within a broader set of context-sensitive modifiers. The comparable performance of LR and EBM indicates that the principal contribution lies in interpretability and cross-cohort comparison rather than predictive superiority.

More importantly, this work addresses three persistent gaps in the literature. First, it moves beyond fragmented descriptions of mid- and late-life depressive risk and provides an integrative framework for organizing predictive correlates. Second, it distinguishes universally shared predictors from context-specific modifiers across countries, thereby clarifying how common risk foundations coexist with cross-national heterogeneity. Third, it demonstrates the value of interpretable analytic strategies for characterizing complex vulnerability structures in aging populations. Taken together, these findings support a two-level logic for screening and prevention: a transferable first-line framework centered on low-cost, cross-culturally stable consistently important cross-sectional correlates, complemented by contextually calibrated signals that refine risk stratification within local populations. In this way, the present study provides a structurally grounded basis for cross-sectional screening and further assessment, stratified prevention, and precision-oriented mental health management among middle-aged and older adults with chronic conditions, while further validation in broader aging populations remains necessary.

## Data Availability

The original contributions presented in the study are included in the article/**Supplementary Material**. Further inquiries can be directed to the corresponding author.
