# Supplementary material for: Cross-national predictive correlates of depressive symptoms among middle-aged and older adults with chronic conditions: an explainable machine learning analysis
Source: Front Psychiatry. 2026 Jul 28;17:1901045. doi: 10.3389/fpsyt.2026.1901045 (PMC13457582; doi:10.3389/fpsyt.2026.1901045)

Supplementary Material

Supplementary Table 1: Descriptive statistical analysis 3

Supplementary Table 2: Descriptive statistical analysis of relevant indicators of CHARLS .. 8

Supplementary Table 3: Descriptive statistical analysis of relevant indicators of ELSA 13

Supplementary Table 4: Descriptive statistical analysis of relevant indicators of HRS 18

Supplementary Table 5: Descriptive statistical analysis of relevant indicators of KLOSA .. 23

Supplementary Table 6: Descriptive statistical analysis of relevant indicators of MHAS 28

Supplementary Table 7: Descriptive statistical analysis of relevant indicators of SHARE ...33

Supplementary Table 8: Performance scores for internally and externally validated ML model of CHARLS 38

Supplementary Table 9: Performance scores for internally and externally validated ML model of ELSA 39

Supplementary Table 10: Performance scores for internally and externally validated ML model of HRS 40

Supplementary Table 11: Performance scores for internally and externally validated ML model of KLoSA 41

Supplementary Table 12: Performance scores for internally and externally validated ML model of MHAS 42

Supplementary Table 13: Performance scores for internally and externally validated ML model of SHARE 43

Supplementary Table 14: Performance scores of the CHARLS ML model using raw data with SMOTE 44

Supplementary Table 15: Performance scores of the ELSA ML model using raw data with SMOTE 45

Supplementary Table 16: Performance scores of the HRS ML model using raw data with SMOTE 46

Supplementary Table 17: Performance scores of the KLoSA ML model using raw data with SMOT 47

Supplementary Table 18: Performance scores of the MHAS ML model using raw data with SMOTE48

Supplementary Table 19: Performance scores of the SHARE ML model using raw data with SMOT 49

Supplementary Table 20: Indicators meaning and description 50

Supplementary Table 21: Hyperparameter settings for the optimal model in each of the six surveys 53

Supplementary Figure 1: Flow chart of participants selected in the CHARLS 54

Supplementary Figure 2: Flow chart of participants selected in the ELSA 55

Supplementary Figure 3: Flow chart of participants selected in the HRS 56

Supplementary Figure 4: Flow chart of participants selected in the KLoSA 57

Supplementary Figure 5: Flow chart of participants selected in the MHAS 58

Supplementary Figure 6: Flow chart of participants selected in the SHARE 59

Supplementary Figure 7: Cross-national comparison of mean contributions for key predictors across six cohorts 60

1 Supplementary Table 1: Descriptive statistical analysis

Variable	Overall	Depression	Non-depression	Statistic	P-Value
age	84140	21960	62180		
	(100.00%)	(26.10%)	(73.90%)		
age = 1	17117	4318	12799	151.199	0.000
	(20.34%)	(19.66%)	(20.58%)		
age = 2	65449	17022	48427		
	(77.79%)	(77.51%)	(77.88%)		
age = 3	1574	620	954		
	(1.87%)	(2.82%)	(1.53%)		
gender	84140	21960	62180		
	(100.00%)	(26.10%)	(73.90%)		
gender = 0	47190	14534	32656	1229.914	0.000
	(56.09%)	(66.18%)	(52.52%)		
gender = 1	36950	7426	29524		
	(43.91%)	(33.82%)	(47.48%)		
marital status	84140	21960	62180		
	(100.00%)	(26.10%)	(73.90%)		
marital status = 0	28916	9345	19571	882.773	0.000
	(34.37%)	(42.55%)	(31.47%)		
marital status = 1	55224	12615	42609		
	(65.63%)	(57.45%)	(68.53%)		
education level	84140	21960	62180		
	(100.00%)	(26.10%)	(73.90%)		
education level = 1	37344	12322	25022	1750.227	0.000
	(44.38%)	(56.11%)	(40.24%)		
education level = 2	30407	6703	23704		
	(36.14%)	(30.52%)	(38.12%)		
education level = 3	16389	2935	13454		
	(19.48%)	(13.37%)	(21.64%)		
household wealth	84140	21960	62180		
	(100.00%)	(26.10%)	(73.90%)		
household wealth = 1	21020	4078	16942	1370.106	0.000
	(24.98%)	(18.57%)	(27.25%)		
household wealth = 2	21045	4857	16188		
	(25.01%)	(22.12%)	(26.03%)		
household wealth = 3	20809	5706	15103		

	(24.73%)	(25.98%)	(24.29%)		
household wealth = 4	21266	7319	13947		
	(25.27%)	(33.33%)	(22.43%)		
household income	84140	21960	62180		
	(100.00%)	(26.10%)	(73.90%)		
household income = 1	20952	3866	17086	1634.669	0.000
	(24.90%)	(17.60%)	(27.48%)		
household income = 2	21025	4895	16130		
	(24.99%)	(22.29%)	(25.94%)		
household income = 3	21023	5774	15249		
	(24.99%)	(26.29%)	(24.52%)		
household income = 4	21140	7425	13715		
	(25.12%)	(33.81%)	(22.06%)		
employment status	84140	21960	62180		
	(100.00%)	(26.10%)	(73.90%)		
employment status = 1	24861	5274	19587	1509.827	0.000
	(29.55%)	(24.02%)	(31.50%)		
employment status = 2	14765	5671	9094		
	(17.55%)	(25.82%)	(14.63%)		
employment status = 3	44514	11015	33499		
	(52.90%)	(50.16%)	(53.87%)		
smoking	84140	21960	62180		
	(100.00%)	(26.10%)	(73.90%)		
smoking = 1	47263	12684	34579	88.812	0.000
	(56.17%)	(57.76%)	(55.61%)		
smoking = 2	24770	5928	18842		
	(29.44%)	(26.99%)	(30.30%)		
smoking = 3	12107	3348	8759		
	(14.39%)	(15.25%)	(14.09%)		
drinking	84140	21960	62180		
	(100.00%)	(26.10%)	(73.90%)		
drinking = 0	45177	14135	31042	1361.191	0.000
	(53.69%)	(64.37%)	(49.92%)		
drinking = 1	38963	7825	31138		
	(46.31%)	(35.63%)	(50.08%)		
self-rated health	84140	21960	62180		

	(100.00%)	(26.10%)	(73.90%)		
self-rated health = 1	7316	4307	3009	6333.73	0.000
	(8.70%)	(19.61%)	(4.84%)		
self-rated health = 2	19573	6727	12846		
	(23.26%)	(30.63%)	(20.66%)		
self-rated health = 3	30252	5719	24533		
	(35.95%)	(26.04%)	(39.45%)		
self-rated health = 4	19970	3528	16442		
	(23.73%)	(16.07%)	(26.44%)		
self-rated health = 5	7029	1679	5350		
	(8.35%)	(7.65%)	(8.60%)		
adl	84140	21960	62180		
	(100.00%)	(26.10%)	(73.90%)		
adl = 0	73100	16030	57070	5022.33	0.000
	(86.88%)	(73.00%)	(91.78%)		
adl = 1	11040	5930	5110		
	(13.12%)	(27.00%)	(8.22%)		
iadl	84140	21960	62180		
	(100.00%)	(26.10%)	(73.90%)		
iadl = 0	70020	14620	55400	5892.086	0.000
	(83.22%)	(66.58%)	(89.10%)		
iadl = 1	14120	7340	6780		
	(16.78%)	(33.42%)	(10.90%)		
chronic disease count	84140	21960	62180		
	(100.00%)	(26.10%)	(73.90%)		
chronic disease count = 1	18523	2859	15664	2480.411	0.000
	(22.01%)	(13.02%)	(25.19%)		
chronic disease count = 2	23331	5022	18309		
	(27.73%)	(22.87%)	(29.45%)		
chronic disease count = 3	42286	14079	28207		
	(50.26%)	(64.11%)	(45.36%)		
pain distress	84140	21960	62180		
	(100.00%)	(26.10%)	(73.90%)		
pain distress = 0	45978	7514	38464	5002.163	0.000
	(54.64%)	(34.22%)	(61.86%)		
pain distress = 1	38162	14446	23716		
	(45.36%)	(65.78%)	(38.14%)		

verbal memory	84140	21960	62180		
	(100.00%)	(26.10%)	(73.90%)		
verbal memory = <= -2.5	385	179	206	2185.576	0.000
	(0.46%)	(0.82%)	(0.33%)		
verbal memory = (-2.5, -2]	1367	635	732		
	(1.62%)	(2.89%)	(1.18%)		
verbal memory = (-2, -1.5]	4302	1749	2553		
	(5.11%)	(7.96%)	(4.11%)		
verbal memory = (-1.5, -1]	7390	2699	4691		
	(8.78%)	(12.29%)	(7.54%)		
verbal memory = (-1, -0.5]	11779	3653	8126		
	(14.00%)	(16.63%)	(13.07%)		
verbal memory = (-0.5, 0]	15715	4174	11541		
	(18.68%)	(19.01%)	(18.56%)		
verbal memory = (0, 0.5]	13670	3138	10532		
	(16.25%)	(14.29%)	(16.94%)		
verbal memory = (0.5, 1]	16447	3227	13220		
	(19.55%)	(14.69%)	(21.26%)		
verbal memory = (1, 1.5]	8298	1664	6634		
	(9.86%)	(7.58%)	(10.67%)		
verbal memory = (1.5, 2]	3470	643	2827		
	(4.12%)	(2.93%)	(4.55%)		
verbal memory = (2, 2.5]	1055	162	893		
	(1.25%)	(0.74%)	(1.44%)		
verbal memory = > 2.5	262	37	225		
	(0.31%)	(0.17%)	(0.36%)		
orientation to date	84140	21960	62180		
	(100.00%)	(26.10%)	(73.90%)		
orientation to date = 0	1386	641	745	1301.96	0.000
	(1.65%)	(2.92%)	(1.20%)		
orientation to date = 1	2530	1111	1419		
	(3.01%)	(5.06%)	(2.28%)		
orientation to date = 2	12114	4105	8009		
	(14.40%)	(18.69%)	(12.88%)		
orientation to date = 3	68110	16103	52007		
	(80.95%)	(73.33%)	(83.64%)		

executive function	84140 (100.00%)	21960 (26.10%)	62180 (73.90%)		
executive function = 0	2930 (3.48%)	1305 (5.94%)	1625 (2.61%)	2207.695	0.000
executive function = 1	6862 (8.16%)	2690 (12.25%)	4172 (6.71%)		
executive function = 2	6274 (7.46%)	2213 (10.08%)	4061 (6.53%)		
executive function = 3	10737 (12.76%)	3180 (14.48%)	7557 (12.15%)		
executive function = 4	14621 (17.38%)	3886 (17.70%)	10735 (17.26%)		
executive function = 5	42716 (50.77%)	8686 (39.55%)	34030 (54.73%)		

2 Supplementary Table 2: Descriptive statistical analysis of relevant indicators of CHARLS

Variable	Overall	Depression	Non-depression	Statistic	P-Value
age	6734 (100.00%)	2324 (34.51%)	4410 (65.49%)		
age = 1	2791 (41.45%)	971 (41.78%)	1820 (41.27%)	0.167	0.920
age = 2	3937 (58.46%)	1351 (58.13%)	2586 (58.64%)		
age = 3	6 (0.09%)	2 (0.09%)	4 (0.09%)		
gender	6734 (100.00%)	2324 (34.51%)	4410 (65.49%)		
gender = 0	3061 (45.46%)	1262 (54.30%)	1799 (40.79%)	111.484	0.000
gender = 1	3673 (54.54%)	1062 (45.70%)	2611 (59.21%)		
marital status	6734 (100.00%)	2324 (34.51%)	4410 (65.49%)		
marital status = 0	1150 (17.08%)	509 (21.90%)	641 (14.54%)	57.806	0.000
marital status = 1	5584 (82.92%)	1815 (78.10%)	3769 (85.46%)		
education level	6734 (100.00%)	2324 (34.51%)	4410 (65.49%)		
education level = 1	5632 (83.64%)	2087 (89.80%)	3545 (80.39%)	102.899	0.000
education level = 2	936 (13.90%)	213 (9.17%)	723 (16.39%)		
education level = 3	166 (2.47%)	24 (1.03%)	142 (3.22%)		
household wealth	6734 (100.00%)	2324 (34.51%)	4410 (65.49%)		
household wealth = 1	1683 (24.99%)	371 (15.96%)	1312 (29.75%)	225.761	0.000
household wealth = 2	1684	538	1146		

	(25.01%)	(23.15%)	(25.99%)		
household wealth = 3	1628	621	1007		
	(24.18%)	(26.72%)	(22.83%)		
household wealth = 4	1739	794	945		
	(25.82%)	(34.17%)	(21.43%)		
household income	6734	2324	4410		
	(100.00%)	(34.51%)	(65.49%)		
household income = 1	1683	366	1317	225.283	0.000
	(24.99%)	(15.75%)	(29.86%)		
household income = 2	1684	538	1146		
	(25.01%)	(23.15%)	(25.99%)		
household income = 3	1681	662	1019		
	(24.96%)	(28.49%)	(23.11%)		
household income = 4	1686	758	928		
	(25.04%)	(32.62%)	(21.04%)		
employment status	6734	2324	4410		
	(100.00%)	(34.51%)	(65.49%)		
employment status = 1	4234	1472	2762	97.396	0.000
	(62.87%)	(63.34%)	(62.63%)		
employment status = 2	1460	613	847		
	(21.68%)	(26.38%)	(19.21%)		
employment status = 3	1040	239	801		
	(15.44%)	(10.28%)	(18.16%)		
smoking	6734	2324	4410		
	(100.00%)	(34.51%)	(65.49%)		
smoking = 1	3517	1308	2209	24.012	0.000
	(52.23%)	(56.28%)	(50.09%)		
smoking = 2	1217	374	843		
	(18.07%)	(16.09%)	(19.12%)		
smoking = 3	2000	642	1358		
	(29.70%)	(27.62%)	(30.79%)		
drinking	6734	2324	4410		
	(100.00%)	(34.51%)	(65.49%)		
drinking = 0	4167	1601	2566	73.470	0.000
	(61.88%)	(68.89%)	(58.19%)		
drinking = 1	2567	723	1844		
	(38.12%)	(31.11%)	(41.81%)		

self-rated health	6734	2324	4410		
	(100.00%)	(34.51%)	(65.49%)		
self-rated health = 1	375	260	115	740.939	0.000
	(5.57%)	(11.19%)	(2.61%)		
self-rated health = 2	1222	705	517		
	(18.15%)	(30.34%)	(11.72%)		
self-rated health = 3	3436	1069	2367		
	(51.02%)	(46.00%)	(53.67%)		
self-rated health = 4	878	164	714		
	(13.04%)	(7.06%)	(16.19%)		
self-rated health = 5	823	126	697		
	(12.22%)	(5.42%)	(15.80%)		
adl	6734	2324	4410		
	(100.00%)	(34.51%)	(65.49%)		
adl = 0	5703	1682	4021	413.584	0.000
	(84.69%)	(72.38%)	(91.18%)		
adl = 1	1031	642	389		
	(15.31%)	(27.62%)	(8.82%)		
iadl	6734	2324	4410		
	(100.00%)	(34.51%)	(65.49%)		
iadl = 0	5382	1499	3883	524.518	0.000
	(79.92%)	(64.50%)	(88.05%)		
iadl = 1	1352	825	527		
	(20.08%)	(35.50%)	(11.95%)		
chronic disease count	6734	2324	4410		
	(100.00%)	(34.51%)	(65.49%)		
chronic disease count = 1	1231	272	959	196.695	0.000
	(18.28%)	(11.70%)	(21.75%)		
chronic disease count = 2	1485	402	1083		
	(22.05%)	(17.30%)	(24.56%)		
chronic disease count = 3	4018	1650	2368		
	(59.67%)	(71.00%)	(53.70%)		
pain distress	6734	2324	4410		
	(100.00%)	(34.51%)	(65.49%)		
pain distress = 0	2887	627	2260	364.976	0.000
	(42.87%)	(26.98%)	(51.25%)		

pain distress = 1	3847<br>(57.13%)	1697<br>(73.02%)	2150<br>(48.75%)		
verbal memory	6734<br>(100.00%)	2324<br>(34.51%)	4410<br>(65.49%)		
verbal memory = (-2, -1.5]	261<br>(3.88%)	128<br>(5.51%)	133<br>(3.02%)		
verbal memory = (-1.5, -1]	673<br>(9.99%)	301<br>(12.95%)	372<br>(8.44%)		
verbal memory = (-1, -0.5]	703<br>(10.44%)	290<br>(12.48%)	413<br>(9.37%)		
verbal memory = (-0.5, 0]	1069<br>(15.87%)	449<br>(19.32%)	620<br>(14.06%)		
verbal memory = (0, 0.5]	1324<br>(19.66%)	450<br>(19.36%)	874<br>(19.82%)		
verbal memory = (0.5, 1]	1207<br>(17.92%)	358<br>(15.40%)	849<br>(19.25%)		
verbal memory = (1, 1.5]	862<br>(12.80%)	230<br>(9.90%)	632<br>(14.33%)		
verbal memory = (1.5, 2]	544<br>(8.08%)	110<br>(4.73%)	434<br>(9.84%)		
verbal memory = (2, 2.5]	72<br>(1.07%)	7<br>(0.30%)	65<br>(1.47%)		
verbal memory = > 2.5	19<br>(0.28%)	1<br>(0.04%)	18<br>(0.41%)		
orientation to date	6734<br>(100.00%)	2324<br>(34.51%)	4410<br>(65.49%)		
orientation to date = 0	89<br>(1.32%)	58<br>(2.50%)	31<br>(0.70%)	132.003	0.000
orientation to date = 1	501<br>(7.44%)	250<br>(10.76%)	251<br>(5.69%)		
orientation to date = 2	1819<br>(27.01%)	697<br>(29.99%)	1122<br>(25.44%)		
orientation to date = 3	4325<br>(64.23%)	1319<br>(56.76%)	3006<br>(68.16%)		
executive function	6734<br>(100.00%)	2324<br>(34.51%)	4410<br>(65.49%)		
executive function = 0	125	65	60	165.907	0.000

	(1.86%)	(2.80%)	(1.36%)
executive function = 1	993	469	524
	(14.75%)	(20.18%)	(11.88%)
executive function = 2	583	247	336
	(8.66%)	(10.63%)	(7.62%)
executive function = 3	875	314	561
	(12.99%)	(13.51%)	(12.72%)
executive function = 4	1538	514	1024
	(22.84%)	(22.12%)	(23.22%)
executive function = 5	2620	715	1905
	(38.91%)	(30.77%)	(43.20%)

3 Supplementary Table 3: Descriptive statistical analysis of relevant indicators of ELSA

Variable	Overall	Depression	Non-depression	Statistic	P-Value
age	6158 (100.00%)	1052 (17.08%)	5106 (82.92%)		
age = 1	1242 (20.17%)	236 (22.43%)	1006 (19.70%)	18.009	0.000
age = 2	4835 (78.52%)	790 (75.10%)	4045 (79.22%)		
age = 3	81 (1.32%)	26 (2.47%)	55 (1.08%)		
gender	6158 (100.00%)	1052 (17.08%)	5106 (82.92%)		
gender = 0	3320 (53.91%)	673 (63.97%)	2647 (51.84%)	51.188	0.000
gender = 1	2838 (46.09%)	379 (36.03%)	2459 (48.16%)		
marital status	6158 (100.00%)	1052 (17.08%)	5106 (82.92%)		
marital status = 0	2031 (32.98%)	523 (49.71%)	1508 (29.53%)	159.810	0.000
marital status = 1	4127 (67.02%)	529 (50.29%)	3598 (70.47%)		
education level	6158 (100.00%)	1052 (17.08%)	5106 (82.92%)		
education level = 1	1218 (19.78%)	309 (29.37%)	909 (17.80%)	89.093	0.000
education level = 2	3438 (55.83%)	565 (53.71%)	2873 (56.27%)		
education level = 3	1502 (24.39%)	178 (16.92%)	1324 (25.93%)		
household wealth	6158 (100.00%)	1052 (17.08%)	5106 (82.92%)		
household wealth = 1	1540 (25.01%)	155 (14.73%)	1385 (27.12%)	255.398	0.000
household wealth = 2	1539 (24.99%)	192 (18.25%)	1347 (26.38%)		

household wealth = 3	1538	245	1293		
	(24.98%)	(23.29%)	(25.32%)		
household wealth = 4	1541	460	1081		
	(25.02%)	(43.73%)	(21.17%)		
household income	6158	1052	5106		
	(100.00%)	(17.08%)	(82.92%)		
household income = 1	1540	163	1377	241.120	0.000
	(25.01%)	(15.49%)	(26.97%)		
household income = 2	1538	180	1358		
	(24.98%)	(17.11%)	(26.60%)		
household income = 3	1540	257	1283		
	(25.01%)	(24.43%)	(25.13%)		
household income = 4	1540	452	1088		
	(25.01%)	(42.97%)	(21.31%)		
employment status	6158	1052	5106		
	(100.00%)	(17.08%)	(82.92%)		
employment status = 1	2016	233	1783	248.585	0.000
	(32.74%)	(22.15%)	(34.92%)		
employment status = 2	456	193	263		
	(7.41%)	(18.35%)	(5.15%)		
employment status = 3	3686	626	3060		
	(59.86%)	(59.51%)	(59.93%)		
smoking	6158	1052	5106		
	(100.00%)	(17.08%)	(82.92%)		
smoking = 1	2447	378	2069	39.785	0.000
	(39.74%)	(35.93%)	(40.52%)		
smoking = 2	3152	526	2626		
	(51.19%)	(50.00%)	(51.43%)		
smoking = 3	559	148	411		
	(9.08%)	(14.07%)	(8.05%)		
drinking	6158	1052	5106		
	(100.00%)	(17.08%)	(82.92%)		
drinking = 0	785	221	564	76.933	0.000
	(12.75%)	(21.01%)	(11.05%)		
drinking = 1	5373	831	4542		
	(87.25%)	(78.99%)	(88.95%)		

self-rated health	6158	1052	5106		
	(100.00%)	(17.08%)	(82.92%)		
self-rated health = 1	383	229	154	914.784	0.000
	(6.22%)	(21.77%)	(3.02%)		
self-rated health = 2	1042	341	701		
	(16.92%)	(32.41%)	(13.73%)		
self-rated health = 3	2059	323	1736		
	(33.44%)	(30.70%)	(34.00%)		
self-rated health = 4	1862	130	1732		
	(30.24%)	(12.36%)	(33.92%)		
self-rated health = 5	812	29	783		
	(13.19%)	(2.76%)	(15.33%)		
adl	6158	1052	5106		
	(100.00%)	(17.08%)	(82.92%)		
adl = 0	5251	678	4573	436.000	0.000
	(85.27%)	(64.45%)	(89.56%)		
adl = 1	907	374	533		
	(14.73%)	(35.55%)	(10.44%)		
iadl	6158	1052	5106		
	(100.00%)	(17.08%)	(82.92%)		
iadl = 0	5125	629	4496	497.046	0.000
	(83.23%)	(59.79%)	(88.05%)		
iadl = 1	1033	423	610		
	(16.77%)	(40.21%)	(11.95%)		
chronic disease count	6158	1052	5106		
	(100.00%)	(17.08%)	(82.92%)		
chronic disease count = 1	1686	179	1507	117.081	0.000
	(27.38%)	(17.02%)	(29.51%)		
chronic disease count = 2	1941	292	1649		
	(31.52%)	(27.76%)	(32.30%)		
chronic disease count = 3	2531	581	1950		
	(41.10%)	(55.23%)	(38.19%)		
pain distress	6158	1052	5106		
	(100.00%)	(17.08%)	(82.92%)		
pain distress = 0	3726	359	3367	368.188	0.000
	(60.51%)	(34.13%)	(65.94%)		
pain distress = 1	2432	693	1739		

	(39.49%)	(65.87%)	(34.06%)		
verbal memory	6158	1052	5106		
	(100.00%)	(17.08%)	(82.92%)		
verbal memory = <= -2.5	22	6	16		
	(0.36%)	(0.57%)	(0.31%)		
verbal memory = (-2.5, -2]	87	27	60		
	(1.41%)	(2.57%)	(1.18%)		
verbal memory = (-2, -1.5]	265	65	200		
	(4.30%)	(6.18%)	(3.92%)		
verbal memory = (-1.5, -1]	504	121	383		
	(8.18%)	(11.50%)	(7.50%)		
verbal memory = (-1, -0.5]	422	74	348		
	(6.85%)	(7.03%)	(6.82%)		
verbal memory = (-0.5, 0]	1263	230	1033		
	(20.51%)	(21.86%)	(20.23%)		
verbal memory = (0, 0.5]	1490	246	1244		
	(24.20%)	(23.38%)	(24.36%)		
verbal memory = (0.5, 1]	1200	161	1039		
	(19.49%)	(15.30%)	(20.35%)		
verbal memory = (1, 1.5]	617	89	528		
	(10.02%)	(8.46%)	(10.34%)		
verbal memory = (1.5, 2]	117	8	109		
	(1.90%)	(0.76%)	(2.13%)		
verbal memory = (2, 2.5]	139	20	119		
	(2.26%)	(1.90%)	(2.33%)		
verbal memory = > 2.5	32	5	27		
	(0.52%)	(0.48%)	(0.53%)		
orientation to date	6158	1052	5106		
	(100.00%)	(17.08%)	(82.92%)		
orientation to date = 0	30	7	23	14.662	0.002
	(0.49%)	(0.67%)	(0.45%)		
orientation to date = 1	52	13	39		
	(0.84%)	(1.24%)	(0.76%)		
orientation to date = 2	840	177	663		
	(13.64%)	(16.83%)	(12.98%)		
orientation to date = 3	5236	855	4381		

	(85.03%)	(81.27%)	(85.80%)		
executive function	6158	1052	5106		
	(100.00%)	(17.08%)	(82.92%)		
executive function = 0	27	9	18	49.612	0.000
	(0.44%)	(0.86%)	(0.35%)		
executive function = 1	101	30	71		
	(1.64%)	(2.85%)	(1.39%)		
executive function = 2	194	48	146		
	(3.15%)	(4.56%)	(2.86%)		
executive function = 3	567	117	450		
	(9.21%)	(11.12%)	(8.81%)		
executive function = 4	1179	236	943		
	(19.15%)	(22.43%)	(18.47%)		
executive function = 5	4090	612	3478		
	(66.42%)	(58.17%)	(68.12%)		

4 Supplementary Table 4: Descriptive statistical analysis of relevant indicators of HRS

Variable	Overall	Depression	Non-depression	Statistic	P-Value
age	7761 (100.00%)	1541 (19.86%)	6220 (80.14%)		
age = 1	107 (1.38%)	22 (1.43%)	85 (1.37%)	6.189	0.045
age = 2	7275 (93.74%)	1425 (92.47%)	5850 (94.05%)		
age = 3	379 (4.88%)	94 (6.10%)	285 (4.58%)		
gender	7761 (100.00%)	1541 (19.86%)	6220 (80.14%)		
gender = 0	4621 (59.54%)	1039 (67.42%)	3582 (57.59%)	49.186	0.000
gender = 1	3140 (40.46%)	502 (32.58%)	2638 (42.41%)		
marital status	7761 (100.00%)	1541 (19.86%)	6220 (80.14%)		
marital status = 0	3813 (49.13%)	967 (62.75%)	2846 (45.76%)	142.063	0.000
marital status = 1	3948 (50.87%)	574 (37.25%)	3374 (54.24%)		
education level	7761 (100.00%)	1541 (19.86%)	6220 (80.14%)		
education level = 1	1440 (18.55%)	426 (27.64%)	1014 (16.30%)	168.084	0.000
education level = 2	4508 (58.09%)	909 (58.99%)	3599 (57.86%)		
education level = 3	1813 (23.36%)	206 (13.37%)	1607 (25.84%)		
household wealth	7761 (100.00%)	1541 (19.86%)	6220 (80.14%)		
household wealth = 1	1939 (24.98%)	223 (14.47%)	1716 (27.59%)	226.641	0.000
household wealth = 2	1940	315	1625		

	(25.00%)	(20.44%)	(26.13%)		
household wealth = 3	1935	423	1512		
	(24.93%)	(27.45%)	(24.31%)		
household wealth = 4	1947	580	1367		
	(25.09%)	(37.64%)	(21.98%)		
household income	7761	1541	6220		
	(100.00%)	(19.86%)	(80.14%)		
household income = 1	1940	219	1721	290.192	0.000
	(25.00%)	(14.21%)	(27.67%)		
household income = 2	1938	302	1636		
	(24.97%)	(19.60%)	(26.30%)		
household income = 3	1941	400	1541		
	(25.01%)	(25.96%)	(24.77%)		
household income = 4	1942	620	1322		
	(25.02%)	(40.23%)	(21.25%)		
employment status	7761	1541	6220		
	(100.00%)	(19.86%)	(80.14%)		
employment status = 1	1423	173	1250	206.417	0.000
	(18.34%)	(11.23%)	(20.10%)		
employment status = 2	1471	477	994		
	(18.95%)	(30.95%)	(15.98%)		
employment status = 3	4867	891	3976		
	(62.71%)	(57.82%)	(63.92%)		
smoking	7761	1541	6220		
	(100.00%)	(19.86%)	(80.14%)		
smoking = 1	3572	652	2920	50.927	0.000
	(46.02%)	(42.31%)	(46.95%)		
smoking = 2	3544	693	2851		
	(45.66%)	(44.97%)	(45.84%)		
smoking = 3	645	196	449		
	(8.31%)	(12.72%)	(7.22%)		
drinking	7761	1541	6220		
	(100.00%)	(19.86%)	(80.14%)		
drinking = 0	3850	912	2938	70.045	0.000
	(49.61%)	(59.18%)	(47.23%)		
drinking = 1	3911	629	3282		
	(50.39%)	(40.82%)	(52.77%)		

self-rated health	7761	1541	6220		
	(100.00%)	(19.86%)	(80.14%)		
self-rated health = 1	554	322	232	1010.718	0.000
	(7.14%)	(20.90%)	(3.73%)		
self-rated health = 2	1791	602	1189		
	(23.08%)	(39.07%)	(19.12%)		
self-rated health = 3	2726	408	2318		
	(35.12%)	(26.48%)	(37.27%)		
self-rated health = 4	2191	172	2019		
	(28.23%)	(11.16%)	(32.46%)		
self-rated health = 5	499	37	462		
	(6.43%)	(2.40%)	(7.43%)		
adl	7761	1541	6220		
	(100.00%)	(19.86%)	(80.14%)		
adl = 0	6101	810	5291	773.951	0.000
	(78.61%)	(52.56%)	(85.06%)		
adl = 1	1660	731	929		
	(21.39%)	(47.44%)	(14.94%)		
iadl	7761	1541	6220		
	(100.00%)	(19.86%)	(80.14%)		
iadl = 0	6353	931	5422	593.509	0.000
	(81.86%)	(60.42%)	(87.17%)		
iadl = 1	1408	610	798		
	(18.14%)	(39.58%)	(12.83%)		
chronic disease count	7761	1541	6220		
	(100.00%)	(19.86%)	(80.14%)		
chronic disease count = 1	479	43	436	100.999	0.000
	(6.17%)	(2.79%)	(7.01%)		
chronic disease count = 2	1329	169	1160		
	(17.12%)	(10.97%)	(18.65%)		
chronic disease count = 3	5953	1329	4624		
	(76.70%)	(86.24%)	(74.34%)		
pain distress	7761	1541	6220		
	(100.00%)	(19.86%)	(80.14%)		
pain distress = 0	4574	538	4036	457.276	0.000
	(58.94%)	(34.91%)	(64.89%)		

pain distress = 1	3187	1003	2184		
	(41.06%)	(65.09%)	(35.11%)		
verbal memory	7761	1541	6220		
	(100.00%)	(19.86%)	(80.14%)		
verbal memory = <= -2.5	111	35	76		
	(1.43%)	(2.27%)	(1.22%)		
verbal memory = (-2.5, -2]	145	49	96		
	(1.87%)	(3.18%)	(1.54%)		
verbal memory = (-2, -1.5]	484	140	344		
	(6.24%)	(9.09%)	(5.53%)		
verbal memory = (-1.5, -1]	917	246	671		
	(11.82%)	(15.96%)	(10.79%)		
verbal memory = (-1, -0.5]	1441	335	1106		
	(18.57%)	(21.74%)	(17.78%)		
verbal memory = (-0.5, 0]	896	179	717		
	(11.54%)	(11.62%)	(11.53%)		
verbal memory = (0, 0.5]	1678	294	1384		
	(21.62%)	(19.08%)	(22.25%)		
verbal memory = (0.5, 1]	1233	153	1080		
	(15.89%)	(9.93%)	(17.36%)		
verbal memory = (1, 1.5]	561	77	484		
	(7.23%)	(5.00%)	(7.78%)		
verbal memory = (1.5, 2]	204	26	178		
	(2.63%)	(1.69%)	(2.86%)		
verbal memory = (2, 2.5]	57	2	55		
	(0.73%)	(0.13%)	(0.88%)		
verbal memory = > 2.5	34	5	29		
	(0.44%)	(0.32%)	(0.47%)		
orientation to date	7761	1541	6220		
	(100.00%)	(19.86%)	(80.14%)		
orientation to date = 0	172	55	117	81.650	0.000
	(2.22%)	(3.57%)	(1.88%)		
orientation to date = 1	241	78	163		
	(3.11%)	(5.06%)	(2.62%)		
orientation to date = 2	1611	398	1213		
	(20.76%)	(25.83%)	(19.50%)		
orientation to date = 3	5737	1010	4727		

	(73.92%)	(65.54%)	(76.00%)		
executive function	7761	1541	6220		
	(100.00%)	(19.86%)	(80.14%)		
executive function = 0	755	259	496	196.815	0.000
	(9.73%)	(16.81%)	(7.97%)		
executive function = 1	931	256	675		
	(12.00%)	(16.61%)	(10.85%)		
executive function = 2	695	162	533		
	(8.96%)	(10.51%)	(8.57%)		
executive function = 3	1049	190	859		
	(13.52%)	(12.33%)	(13.81%)		
executive function = 4	1428	253	1175		
	(18.40%)	(16.42%)	(18.89%)		
executive function = 5	2903	421	2482		
	(37.40%)	(27.32%)	(39.90%)		

5 Supplementary Table 5: Descriptive statistical analysis of relevant indicators of KLOSA

Variable	Overall	Depression	Non-depression	Statistic	P-Value
age	6704	1445	5259		
	(100.00%)	(21.55%)	(78.45%)		
age = 1	1605	233	1372	90.466	0.000
	(23.94%)	(16.12%)	(26.09%)		
age = 2	4986	1162	3824		
	(74.37%)	(80.42%)	(72.71%)		
age = 3	113	50	63		
	(1.69%)	(3.46%)	(1.20%)		
gender	6704	1445	5259		
	(100.00%)	(21.55%)	(78.45%)		
gender = 0	3880	895	2985	12.254	0.000
	(57.88%)	(61.94%)	(56.76%)		
gender = 1	2824	550	2274		
	(42.12%)	(38.06%)	(43.24%)		
marital status	6704	1445	5259		
	(100.00%)	(21.55%)	(78.45%)		
marital status = 0	1621	516	1105	132.768	0.000
	(24.18%)	(35.71%)	(21.01%)		
marital status = 1	5083	929	4154		
	(75.82%)	(64.29%)	(78.99%)		
education level	6704	1445	5259		
	(100.00%)	(21.55%)	(78.45%)		
education level = 1	3804	975	2829	86.702	0.000
	(56.74%)	(67.47%)	(53.79%)		
education level = 2	2184	349	1835		
	(32.58%)	(24.15%)	(34.89%)		
education level = 3	716	121	595		
	(10.68%)	(8.37%)	(11.31%)		
household wealth	6704	1445	5259		
	(100.00%)	(21.55%)	(78.45%)		
household wealth = 1	1671	293	1378	62.442	0.000
	(24.93%)	(20.28%)	(26.20%)		
household wealth = 2	1679	310	1369		
	(25.04%)	(21.45%)	(26.03%)		

household wealth = 3	1570	353	1217		
	(23.42%)	(24.43%)	(23.14%)		
household wealth = 4	1784	489	1295		
	(26.61%)	(33.84%)	(24.62%)		
household income	6704	1445	5259		
	(100.00%)	(21.55%)	(78.45%)		
household income = 1	1605	242	1363	159.744	0.000
	(23.94%)	(16.75%)	(25.92%)		
household income = 2	1720	309	1411		
	(25.66%)	(21.38%)	(26.83%)		
household income = 3	1635	341	1294		
	(24.39%)	(23.60%)	(24.61%)		
household income = 4	1744	553	1191		
	(26.01%)	(38.27%)	(22.65%)		
employment status	6704	1445	5259		
	(100.00%)	(21.55%)	(78.45%)		
employment status = 1	2292	294	1998	157.392	0.000
	(34.19%)	(20.35%)	(37.99%)		
employment status = 2	46	14	32		
	(0.69%)	(0.97%)	(0.61%)		
employment status = 3	4366	1137	3229		
	(65.13%)	(78.69%)	(61.40%)		
smoking	6704	1445	5259		
	(100.00%)	(21.55%)	(78.45%)		
smoking = 1	4655	1016	3639	4.312	0.116
	(69.44%)	(70.31%)	(69.20%)		
smoking = 2	1323	294	1029		
	(19.73%)	(20.35%)	(19.57%)		
smoking = 3	726	135	591		
	(10.83%)	(9.34%)	(11.24%)		
drinking	6704	1445	5259		
	(100.00%)	(21.55%)	(78.45%)		
drinking = 0	4459	1067	3392	43.996	0.000
	(66.51%)	(73.84%)	(64.50%)		
drinking = 1	2245	378	1867		
	(33.49%)	(26.16%)	(35.50%)		

self-rated health	6704 (100.00%)	1445 (21.55%)	5259 (78.45%)		
self-rated health = 1	1414 (21.09%)	646 (44.71%)	768 (14.60%)	629.321	0.000
self-rated health = 2	2506 (37.38%)	427 (29.55%)	2079 (39.53%)		
self-rated health = 3	2387 (35.61%)	328 (22.70%)	2059 (39.15%)		
self-rated health = 4	371 (5.53%)	42 (2.91%)	329 (6.26%)		
self-rated health = 5	26 (0.39%)	2 (0.14%)	24 (0.46%)		
adl	6704 (100.00%)	1445 (21.55%)	5259 (78.45%)		
adl = 0	6414 (95.67%)	1265 (87.54%)	5149 (97.91%)	291.756	0.000
adl = 1	290 (4.33%)	180 (12.46%)	110 (2.09%)		
iadl	6704 (100.00%)	1445 (21.55%)	5259 (78.45%)		
iadl = 0	5906 (88.10%)	1079 (74.67%)	4827 (91.79%)	314.979	0.000
iadl = 1	798 (11.90%)	366 (25.33%)	432 (8.21%)		
chronic disease count	6704 (100.00%)	1445 (21.55%)	5259 (78.45%)		
chronic disease count = 1	2456 (36.63%)	365 (25.26%)	2091 (39.76%)	186.576	0.000
chronic disease count = 2	2040 (30.43%)	396 (27.40%)	1644 (31.26%)		
chronic disease count = 3	2208 (32.94%)	684 (47.34%)	1524 (28.98%)		
pain distress	6704 (100.00%)	1445 (21.55%)	5259 (78.45%)		
pain distress = 0	2460 (36.69%)	369 (25.54%)	2091 (39.76%)	98.117	0.000
pain distress = 1	4244	1076	3168		

	(63.31%)	(74.46%)	(60.24%)		
verbal memory	6704	1445	5259		
	(100.00%)	(21.55%)	(78.45%)		
verbal memory = <= -2.5	184	104	80		
	(2.74%)	(7.20%)	(1.52%)		
verbal memory = (-2.5, -2]	235	104	131		
	(3.51%)	(7.20%)	(2.49%)		
verbal memory = (-2, -1.5]	441	154	287		
	(6.58%)	(10.66%)	(5.46%)		
verbal memory = (-1, -0.5]	758	265	493		
	(11.31%)	(18.34%)	(9.37%)		
verbal memory = (-0.5, 0]	1203	300	903		
	(17.94%)	(20.76%)	(17.17%)		
verbal memory = (0, 0.5]	1440	222	1218		
	(21.48%)	(15.36%)	(23.16%)		
verbal memory = (0.5, 1]	2443	296	2147		
	(36.44%)	(20.48%)	(40.83%)		
orientation to date	6704	1445	5259		
	(100.00%)	(21.55%)	(78.45%)		
orientation to date = 0	118	60	58	424.817	0.000
	(1.76%)	(4.15%)	(1.10%)		
orientation to date = 1	329	173	156		
	(4.91%)	(11.97%)	(2.97%)		
orientation to date = 2	620	243	377		
	(9.25%)	(16.82%)	(7.17%)		
orientation to date = 3	5637	969	4668		
	(84.08%)	(67.06%)	(88.76%)		
executive function	6704	1445	5259		
	(100.00%)	(21.55%)	(78.45%)		
executive function = 0	632	297	335	474.525	0.000
	(9.43%)	(20.55%)	(6.37%)		
executive function = 1	395	129	266		
	(5.89%)	(8.93%)	(5.06%)		
executive function = 2	421	142	279		
	(6.28%)	(9.83%)	(5.31%)		
executive function = 3	956	246	710		

	(14.26%)	(17.02%)	(13.50%)
executive function = 4	711	165	546
	(10.61%)	(11.42%)	(10.38%)
executive function = 5	3589	466	3123
	(53.54%)	(32.25%)	(59.38%)

6 Supplementary Table 6: Descriptive statistical analysis of relevant indicators of MHAS

Variable	Overall	Depression	Non-depression	Statistic	P-Value
age	13007 (100.00%)	4013 (30.85%)	8994 (69.15%)		
age = 1	3572 (27.46%)	940 (23.42%)	2632 (29.26%)	74.869	0.000
age = 2	9264 (71.22%)	2986 (74.41%)	6278 (69.80%)		
age = 3	171 (1.31%)	87 (2.17%)	84 (0.93%)		
gender	13007 (100.00%)	4013 (30.85%)	8994 (69.15%)		
gender = 0	7497 (57.64%)	2751 (68.55%)	4746 (52.77%)	282.478	0.000
gender = 1	5510 (42.36%)	1262 (31.45%)	4248 (47.23%)		
marital status	13007 (100.00%)	4013 (30.85%)	8994 (69.15%)		
marital status = 0	5142 (39.53%)	1897 (47.27%)	3245 (36.08%)	144.932	0.000
marital status = 1	7865 (60.47%)	2116 (52.73%)	5749 (63.92%)		
education level	13007 (100.00%)	4013 (30.85%)	8994 (69.15%)		
education level = 1	11146 (85.69%)	3746 (93.35%)	7400 (82.28%)	278.308	0.000
education level = 2	470 (3.61%)	76 (1.89%)	394 (4.38%)		
education level = 3	1391 (10.69%)	191 (4.76%)	1200 (13.34%)		
household wealth	13007 (100.00%)	4013 (30.85%)	8994 (69.15%)		
household wealth = 1	3243 (24.93%)	866 (21.58%)	2377 (26.43%)	70.475	0.000
household wealth = 2	3260	931	2329		

	(25.06%)	(23.20%)	(25.90%)		
household wealth = 3	3200	1050	2150		
	(24.60%)	(26.16%)	(23.90%)		
household wealth = 4	3304	1166	2138		
	(25.40%)	(29.06%)	(23.77%)		
household income	13007	4013	8994		
	(100.00%)	(30.85%)	(69.15%)		
household income = 1	3251	657	2594	258.360	0.000
	(24.99%)	(16.37%)	(28.84%)		
household income = 2	3191	985	2206		
	(24.53%)	(24.55%)	(24.53%)		
household income = 3	3292	1202	2090		
	(25.31%)	(29.95%)	(23.24%)		
household income = 4	3273	1169	2104		
	(25.16%)	(29.13%)	(23.39%)		
employment status	13007	4013	8994		
	(100.00%)	(30.85%)	(69.15%)		
employment status = 1	4989	1158	3831	362.347	0.000
	(38.36%)	(28.86%)	(42.60%)		
employment status = 2	6282	2439	3843		
	(48.30%)	(60.78%)	(42.73%)		
employment status = 3	1736	416	1320		
	(13.35%)	(10.37%)	(14.68%)		
smoking	13007	4013	8994		
	(100.00%)	(30.85%)	(69.15%)		
smoking = 1	7758	2571	5187	48.035	0.000
	(59.64%)	(64.07%)	(57.67%)		
smoking = 2	3730	1039	2691		
	(28.68%)	(25.89%)	(29.92%)		
smoking = 3	1519	403	1116		
	(11.68%)	(10.04%)	(12.41%)		
drinking	13007	4013	8994		
	(100.00%)	(30.85%)	(69.15%)		
drinking = 0	9962	3343	6619	145.398	0.000
	(76.59%)	(83.30%)	(73.59%)		
drinking = 1	3045	670	2375		
	(23.41%)	(16.70%)	(26.41%)		

self-rated health	13007 (100.00%)	4013 (30.85%)	8994 (69.15%)		
self-rated health = 1	309 (2.38%)	24 (0.60%)	285 (3.17%)	1593.258	0.000
self-rated health = 2	429 (3.30%)	24 (0.60%)	405 (4.50%)		
self-rated health = 3	3435 (26.41%)	521 (12.98%)	2914 (32.40%)		
self-rated health = 4	6929 (53.27%)	2223 (55.39%)	4706 (52.32%)		
self-rated health = 5	1905 (14.65%)	1221 (30.43%)	684 (7.61%)		
adl	13007 (100.00%)	4013 (30.85%)	8994 (69.15%)		
adl = 0	10577 (81.32%)	2612 (65.09%)	7965 (88.56%)	1004.641	0.000
adl = 1	2430 (18.68%)	1401 (34.91%)	1029 (11.44%)		
iadl	13007 (100.00%)	4013 (30.85%)	8994 (69.15%)		
iadl = 0	11282 (86.74%)	2989 (74.48%)	8293 (92.21%)	756.161	0.000
iadl = 1	1725 (13.26%)	1024 (25.52%)	701 (7.79%)		
chronic disease count	13007 (100.00%)	4013 (30.85%)	8994 (69.15%)		
chronic disease count = 1	3175 (24.41%)	538 (13.41%)	2637 (29.32%)	599.834	0.000
chronic disease count = 2	4071 (31.30%)	1105 (27.54%)	2966 (32.98%)		
chronic disease count = 3	5761 (44.29%)	2370 (59.06%)	3391 (37.70%)		
pain distress	13007 (100.00%)	4013 (30.85%)	8994 (69.15%)		
pain distress = 0	8006 (61.55%)	1622 (40.42%)	6384 (70.98%)	1093.902	0.000

pain distress = 1	5001	2391	2610		
	(38.45%)	(59.58%)	(29.02%)		
verbal memory	13007	4013	8994		
	(100.00%)	(30.85%)	(69.15%)		
verbal memory = <= -2.5	68	34	34		
	(0.52%)	(0.85%)	(0.38%)		
verbal memory = (-2.5, -2]	313	132	181		
	(2.41%)	(3.29%)	(2.01%)		
verbal memory = (-2, -1.5]	781	317	464		
	(6.00%)	(7.90%)	(5.16%)		
verbal memory = (-1.5, -1]	1216	464	752		
	(9.35%)	(11.56%)	(8.36%)		
verbal memory = (-1, -0.5]	1663	592	1071		
	(12.79%)	(14.75%)	(11.91%)		
verbal memory = (-0.5, 0]	1859	598	1261		
	(14.29%)	(14.90%)	(14.02%)		
verbal memory = (0, 0.5]	2781	810	1971		
	(21.38%)	(20.18%)	(21.91%)		
verbal memory = (0.5, 1]	2050	540	1510		
	(15.76%)	(13.46%)	(16.79%)		
verbal memory = (1, 1.5]	1616	382	1234		
	(12.42%)	(9.52%)	(13.72%)		
verbal memory = (1.5, 2]	605	132	473		
	(4.65%)	(3.29%)	(5.26%)		
verbal memory = (2, 2.5]	55	12	43		
	(0.42%)	(0.30%)	(0.48%)		
orientation to date	13007	4013	8994		
	(100.00%)	(30.85%)	(69.15%)		
orientation to date = 0	762	355	407	210.198	0.000
	(5.86%)	(8.85%)	(4.53%)		
orientation to date = 1	1002	406	596		
	(7.70%)	(10.12%)	(6.63%)		
orientation to date = 2	3199	1094	2105		
	(24.59%)	(27.26%)	(23.40%)		
orientation to date = 3	8044	2158	5886		
	(61.84%)	(53.78%)	(65.44%)		
executive function	13007	4013	8994		

	(100.00%)	(30.85%)	(69.15%)		
executive function = 0	751	312	439	190.496	0.000
	(5.77%)	(7.77%)	(4.88%)		
executive function = 1	2018	739	1279		
	(15.51%)	(18.42%)	(14.22%)		
executive function = 2	2167	754	1413		
	(16.66%)	(18.79%)	(15.71%)		
executive function = 3	2478	799	1679		
	(19.05%)	(19.91%)	(18.67%)		
executive function = 4	2429	682	1747		
	(18.67%)	(16.99%)	(19.42%)		
executive function = 5	3164	727	2437		
	(24.33%)	(18.12%)	(27.10%)		

7 Supplementary Table 7: Descriptive statistical analysis of relevant indicators of SHARE

Variable	Overall	Depression	Non-depression	Statistic	P-Value
age	43776	11585	32191		
	(100.00%)	(26.46%)	(73.54%)		
age = 1	7800	1916	5884	141.881	0.000
	(17.82%)	(16.54%)	(18.28%)		
age = 2	35152	9308	25844		
	(80.30%)	(80.35%)	(80.28%)		
age = 3	824	361	463		
	(1.88%)	(3.12%)	(1.44%)		
gender	43776	11585	32191		
	(100.00%)	(26.46%)	(73.54%)		
gender = 0	24811	7914	16897	867.972	0.000
	(56.68%)	(68.31%)	(52.49%)		
gender = 1	18965	3671	15294		
	(43.32%)	(31.69%)	(47.51%)		
marital status	43776	11585	32191		
	(100.00%)	(26.46%)	(73.54%)		
marital status = 0	15159	4933	10226	439.638	0.000
	(34.63%)	(42.58%)	(31.77%)		
marital status = 1	28617	6652	21965		
	(65.37%)	(57.42%)	(68.23%)		
education level	43776	11585	32191		
	(100.00%)	(26.46%)	(73.54%)		
education level = 1	14104	4779	9325	640.175	0.000
	(32.22%)	(41.25%)	(28.97%)		
education level = 2	18871	4591	14280		
	(43.11%)	(39.63%)	(44.36%)		
education level = 3	10801	2215	8586		
	(24.67%)	(19.12%)	(26.67%)		
household wealth	43776	11585	32191		
	(100.00%)	(26.46%)	(73.54%)		
household wealth = 1	10944	2170	8774	711.431	0.000
	(25.00%)	(18.73%)	(27.26%)		
household wealth = 2	10943	2571	8372		
	(25.00%)	(22.19%)	(26.01%)		

household wealth = 3	10938	3014	7924		
	(24.99%)	(26.02%)	(24.62%)		
household wealth = 4	10951	3830	7121		
	(25.02%)	(33.06%)	(22.12%)		
household income	43776	11585	32191		
	(100.00%)	(26.46%)	(73.54%)		
household income = 1	10933	2219	8714	706.132	0.000
	(24.97%)	(19.15%)	(27.07%)		
household income = 2	10954	2581	8373		
	(25.02%)	(22.28%)	(26.01%)		
household income = 3	10934	2912	8022		
	(24.98%)	(25.14%)	(24.92%)		
household income = 4	10955	3873	7082		
	(25.03%)	(33.43%)	(22.00%)		
employment status	43776	11585	32191		
	(100.00%)	(26.46%)	(73.54%)		
employment status = 1	9907	1944	7963	603.969	0.000
	(22.63%)	(16.78%)	(24.74%)		
employment status = 2	5050	1935	3115		
	(11.54%)	(16.70%)	(9.68%)		
employment status = 3	28819	7706	21113		
	(65.83%)	(66.52%)	(65.59%)		
smoking	43776	11585	32191		
	(100.00%)	(26.46%)	(73.54%)		
smoking = 1	25314	6759	18555	10.157	0.006
	(57.83%)	(58.34%)	(57.64%)		
smoking = 2	11804	3002	8802		
	(26.96%)	(25.91%)	(27.34%)		
smoking = 3	6658	1824	4834		
	(15.21%)	(15.74%)	(15.02%)		
drinking	43776	11585	32191		
	(100.00%)	(26.46%)	(73.54%)		
drinking = 0	21954	6991	14963	654.374	0.000
	(50.15%)	(60.35%)	(46.48%)		
drinking = 1	21822	4594	17228		
	(49.85%)	(39.65%)	(53.52%)		

self-rated health	43776 (100.00%)	11585 (26.46%)	32191 (73.54%)		
self-rated health = 1	4281 (9.78%)	2826 (24.39%)	1455 (4.52%)	6108.995	0.000
self-rated health = 2	12583 (28.74%)	4628 (39.95%)	7955 (24.71%)		
self-rated health = 3	16209 (37.03%)	3070 (26.50%)	13139 (40.82%)		
self-rated health = 4	7739 (17.68%)	797 (6.88%)	6942 (21.57%)		
self-rated health = 5	2964 (6.77%)	264 (2.28%)	2700 (8.39%)		
adl	43776 (100.00%)	11585 (26.46%)	32191 (73.54%)		
adl = 0	39054 (89.21%)	8983 (77.54%)	30071 (93.41%)	2229.193	0.000
adl = 1	4722 (10.79%)	2602 (22.46%)	2120 (6.59%)		
iadl	43776 (100.00%)	11585 (26.46%)	32191 (73.54%)		
iadl = 0	35972 (82.17%)	7493 (64.68%)	28479 (88.47%)	3289.823	0.000
iadl = 1	7804 (17.83%)	4092 (35.32%)	3712 (11.53%)		
chronic disease count	43776 (100.00%)	11585 (26.46%)	32191 (73.54%)		
chronic disease count = 1	9496 (21.69%)	1462 (12.62%)	8034 (24.96%)	1441.255	0.000
chronic disease count = 2	12465 (28.47%)	2658 (22.94%)	9807 (30.47%)		
chronic disease count = 3	21815 (49.83%)	7465 (64.44%)	14350 (44.58%)		
pain distress	43776 (100.00%)	11585 (26.46%)	32191 (73.54%)		
pain distress = 0	24325 (55.57%)	3999 (34.52%)	20326 (63.14%)	2825.706	0.000
pain distress = 1	19451	7586	11865		

	(44.43%)	(65.48%)	(36.86%)		
verbal memory	43776	11585	32191		
	(100.00%)	(26.46%)	(73.54%)		
verbal memory = (-2.5, -2]	587	323	264		
	(1.34%)	(2.79%)	(0.82%)		
verbal memory = (-2, -1.5]	2070	945	1125		
	(4.73%)	(8.16%)	(3.49%)		
verbal memory = (-1.5, -1]	4080	1567	2513		
	(9.32%)	(13.53%)	(7.81%)		
verbal memory = (-1, -0.5]	6792	2097	4695		
	(15.52%)	(18.10%)	(14.58%)		
verbal memory = (-0.5, 0]	9425	2418	7007		
	(21.53%)	(20.87%)	(21.77%)		
verbal memory = (0, 0.5]	4957	1116	3841		
	(11.32%)	(9.63%)	(11.93%)		
verbal memory = (0.5, 1]	8314	1719	6595		
	(18.99%)	(14.84%)	(20.49%)		
verbal memory = (1, 1.5]	4642	886	3756		
	(10.60%)	(7.65%)	(11.67%)		
verbal memory = (1.5, 2]	2000	367	1633		
	(4.57%)	(3.17%)	(5.07%)		
verbal memory = (2, 2.5]	732	121	611		
	(1.67%)	(1.04%)	(1.90%)		
verbal memory = > 2.5	177	26	151		
	(0.40%)	(0.22%)	(0.47%)		
orientation to date	43776	11585	32191		
	(100.00%)	(26.46%)	(73.54%)		
orientation to date = 0	215	106	109	425.445	0.000
	(0.49%)	(0.91%)	(0.34%)		
orientation to date = 1	405	191	214		
	(0.93%)	(1.65%)	(0.66%)		
orientation to date = 2	4025	1496	2529		
	(9.19%)	(12.91%)	(7.86%)		
orientation to date = 3	39131	9792	29339		
	(89.39%)	(84.52%)	(91.14%)		
executive function	43776	11585	32191		

	(100.00%)	(26.46%)	(73.54%)		
executive function = 0	640	363	277	1221.475	0.000
	(1.46%)	(3.13%)	(0.86%)		
executive function = 1	2424	1067	1357		
	(5.54%)	(9.21%)	(4.22%)		
executive function = 2	2214	860	1354		
	(5.06%)	(7.42%)	(4.21%)		
executive function = 3	4812	1514	3298		
	(10.99%)	(13.07%)	(10.25%)		
executive function = 4	7336	2036	5300		
	(16.76%)	(17.57%)	(16.46%)		
executive function = 5	26350	5745	20605		
	(60.19%)	(49.59%)	(64.01%)		

8 Supplementary Table 8: Performance scores for internally and externally validated ML model of CHARLS

Model	AUC	Accuracy	Precision	Recall	F score
LogisticRegression	0.7636(0.7367–0.7908)	0.7016 (0.6756–0.7261)	0.5537 (0.5119–0.5936)	0.6989 (0.6571–0.7404)	0.6179 (0.5813–0.6530)
RandomForest	0.7524(0.7445–0.7985)	0.7350 (0.7097–0.7573)	0.6280 (0.5810–0.6731)	0.5699 (0.5244–0.6137)	0.5975 (0.5576–0.6331)
XGBoost	0.7032(0.6904–0.7379)	0.7320 (0.7075–0.7558)	0.6429 (0.5934–0.6906)	0.5032 (0.4557–0.5470)	0.5645 (0.5222–0.6041)
SVM	0.7428(0.7128–0.7702)	0.6889 (0.6637–0.7142)	0.5429 (0.5000–0.5843)	0.6258 (0.5802–0.6695)	0.5814 (0.5445–0.6166)
MLP	0.6806(0.6609–0.7201)	0.6852 (0.6600–0.7097)	0.5455 (0.5000–0.5924)	0.5290 (0.4853–0.5729)	0.5371 (0.4994–0.5745)
EBM	0.7659(0.7371–0.7914)	0.7231 (0.6986–0.7461)	0.6299 (0.5763–0.6794)	0.4796 (0.4335–0.5253)	0.5446 (0.5000–0.5838)

9 Supplementary Table 9: Performance scores for internally and externally validated ML model of ELSA

Model	AUC	Accuracy	Precision	Recall	F score
LogisticRegression	0.8032(0.7708–0.8360)	0.7500 (0.7256–0.7760)	0.3763 (0.3285–0.4247)	0.7095 (0.6517–0.7685)	0.4917 (0.4411–0.5393)
RandomForest	0.7737(0.7643–0.8302)	0.8409 (0.8206–0.8604)	0.5437 (0.4685–0.6185)	0.4143 (0.3472–0.4800)	0.4703 (0.4079–0.5297)
XGBoost	0.7256(0.7060–0.7356)	0.8450 (0.8247–0.8644)	0.5905 (0.4955–0.6863)	0.2952 (0.2331–0.3575)	0.3937 (0.3224–0.4587)
SVM	0.7008(0.6875–0.7636)	0.7687 (0.7459–0.7914)	0.3737 (0.3168–0.4286)	0.5286 (0.4608–0.5961)	0.4379 (0.3800–0.4916)
MLP	0.6699(0.6352–0.7164)	0.7662 (0.7435–0.7890)	0.3427 (0.2846–0.4047)	0.4048 (0.3395–0.4740)	0.3712 (0.3153–0.4280)
EBM	0.8029(0.7718–0.8361)	0.8515 (0.8320–0.8701)	0.6484 (0.5526–0.7419)	0.2810 (0.2232–0.3404)	0.3920 (0.3223–0.4578)

10 Supplementary Table 10: Performance scores for internally and externally validated ML model of HRS

Model	AUC	Accuracy	Precision	Recall	F score
LogisticRegression	0.7915(0.7619–0.8167)	0.7386 (0.7173–0.7611)	0.4099 (0.3683–0.4539)	0.7240 (0.6766–0.7719)	0.5235 (0.4826–0.5647)
RandomForest	0.7599(0.7362–0.7922)	0.8062 (0.7862–0.8255)	0.5117 (0.4510–0.5686)	0.4968 (0.4365–0.5535)	0.5041 (0.4494–0.5517)
XGBoost	0.685(0.6668–0.7176)	0.8126 (0.7933–0.8313)	0.5563 (0.4768–0.6328)	0.2727 (0.2226–0.3232)	0.3660 (0.3083–0.4191)
SVM	0.7389(0.7172–0.7823)	0.7502 (0.7283–0.7721)	0.4119 (0.3663–0.4580)	0.6071 (0.5512–0.6626)	0.4908 (0.4435–0.5343)
MLP	0.6778(0.6462–0.7123)	0.7418 (0.7186–0.7643)	0.3524 (0.3000–0.4046)	0.3604 (0.3062–0.4136)	0.3563 (0.3072–0.4033)
EBM	0.7941(0.7631–0.8192)	0.8158 (0.7952–0.8345)	0.5797 (0.4931–0.6642)	0.2597 (0.2122–0.3077)	0.3587 (0.3002–0.4132)

11 Supplementary Table 11: Performance scores for internally and externally validated ML model of KLoSA

Model	AUC	Accuracy	Precision	Recall	F score
LogisticRegression	0.7559(0.7261–0.7915)	0.7151 (0.6905–0.7375)	0.4017 (0.3573–0.4475)	0.6574 (0.6028–0.7148)	0.4987 (0.4543–0.5452)
RandomForest	0.7534(0.7319–0.7942)	0.8106 (0.7897–0.8315)	0.5778 (0.5118–0.6429)	0.4498 (0.3935–0.5087)	0.5058 (0.4507–0.5587)
XGBoost	0.7005(0.6853–0.7481)	0.8136 (0.7927–0.8337)	0.6535 (0.5690–0.7348)	0.2872 (0.2376–0.3415)	0.3990 (0.3413–0.4587)
SVM	0.7115(0.6904–0.7479)	0.7293 (0.7047–0.7532)	0.4111 (0.3632–0.4580)	0.5917 (0.5342–0.6482)	0.4851 (0.4389–0.5300)
MLP	0.6318(0.6131–0.6973)	0.7062 (0.6823–0.7301)	0.3569 (0.3089–0.4085)	0.4533 (0.3951–0.5119)	0.3994 (0.3516–0.4475)
EBM	0.7762(0.7447–0.8075)	0.8143 (0.7942–0.8345)	0.6562 (0.5769–0.7387)	0.2907 (0.2388–0.3420)	0.4029 (0.3418–0.4615)

12 Supplementary Table 12: Performance scores for internally and externally validated ML model of MHAS

Model	AUC	Accuracy	Precision	Recall	F score
LogisticRegression	0.7712(0.7517–0.7911)	0.7098 (0.6925–0.7283)	0.5226 (0.4923–0.5535)	0.6899 (0.6572–0.7213)	0.5947 (0.5681–0.6221)
RandomForest	0.7471(0.7278–0.7772)	0.7283 (0.7118–0.7460)	0.5584 (0.5242–0.5933)	0.5716 (0.5353–0.6054)	0.5649 (0.5344–0.5943)
XGBoost	0.7076(0.6861–0.7360)	0.7325 (0.7152–0.7498)	0.5989 (0.5577–0.6403)	0.4035 (0.3684–0.4395)	0.4821 (0.4474–0.5150)
SVM	0.7532(0.7202–0.7619)	0.7022 (0.6856–0.7198)	0.5137 (0.4829–0.5442)	0.6550 (0.6218–0.6883)	0.5758 (0.5479–0.6027)
MLP	0.6941(0.6767–0.7190)	0.6822 (0.6645–0.6998)	0.4878 (0.4559–0.5194)	0.5953 (0.5603–0.6286)	0.5362 (0.5069–0.5632)
EBM	0.7734(0.7509–0.7905)	0.7444 (0.7271–0.7617)	0.6312 (0.5896–0.6732)	0.4134 (0.3784–0.4485)	0.4996 (0.4656–0.5328)

13 Supplementary Table 13: Performance scores for internally and externally validated ML model of SHARE

Model	AUC	Accuracy	Precision	Recall	F score
LogisticRegression	0.7608(0.7385–0.7711)	0.6978 (0.6880–0.7069)	0.4527 (0.4354–0.4692)	0.6798 (0.6604–0.6985)	0.5435 (0.5272–0.5586)
RandomForest	0.7303(0.7177–0.7603)	0.7457 (0.7368–0.7548)	0.5190 (0.4979–0.5388)	0.5300 (0.5090–0.5501)	0.5245 (0.5056–0.5416)
XGBoost	0.7298(0.7111–0.7635)	0.7723 (0.7632–0.7808)	0.6146 (0.5882–0.6396)	0.3738 (0.3529–0.3937)	0.4648 (0.4443–0.4847)
SVM	0.7212(0.7197–0.7535)	0.6992 (0.6895–0.7080)	0.4530 (0.4355–0.4698)	0.6586 (0.6387–0.6776)	0.5368 (0.5202–0.5520)
MLP	0.6984(0.6748–0.7298)	0.7020 (0.6924–0.7112)	0.4509 (0.4334–0.4685)	0.5783 (0.5578–0.5981)	0.5067 (0.4902–0.5228)
EBM	0.7653(0.7541–0.7764)	0.7771 (0.7680–0.7853)	0.6373 (0.6107–0.6633)	0.3656 (0.3454–0.3854)	0.4646 (0.4438–0.4846)

14 Supplementary Table 14: Performance scores of the CHARLS ML model using raw data with SMOTE

Model	AUC	Accuracy	Precision	Recall	F score
RandomForest	0.7720(0.7620–0.7972)	0.7259 (0.7120–0.7572)	0.6370 (0.5905–0.6643)	0.5789 (0.5226–0.6129)	0.6066 (0.5608–0.6331)
EBM	0.7662(0.7420–0.7850)	0.7333 (0.7120–0.7550)	0.6533 (0.6100–0.6949)	0.4673 (0.4452–0.5333)	0.5449 (0.5185–0.5988)
LogisticRegression	0.7650(0.7471–0.7968)	0.6936 (0.6771–0.7268)	0.5477 (0.5254–0.5845)	0.6889 (0.6559–0.7398)	0.6102 (0.5865–0.6473)
XGBoost	0.7505(0.7390–0.7828)	0.7366 (0.7090–0.7528)	0.6339 (0.6005–0.6839)	0.5122 (0.4581–0.5462)	0.5666 (0.5236–0.6005)
SVM	0.7411(0.7244–0.7527)	0.6779 (0.6644–0.7127)	0.5559 (0.5123–0.5749)	0.6138 (0.5806–0.6688)	0.5834 (0.5491–0.6131)
MLP	0.7001(0.6907–0.7290)	0.6752 (0.6607–0.7090)	0.5385 (0.5086–0.5824)	0.5340 (0.4838–0.5742)	0.5362 (0.4983–0.5729)

15 Supplementary Table 15: Performance scores of the ELSA ML model using raw data with SMOTE

Model	AUC	Accuracy	Precision	Recall	F score
EBM	0.8059(0.7796–0.8265)	0.8433 (0.8385–0.8661)	0.6941 (0.5625–0.7558)	0.2922 (0.2190–0.3381)	0.4113 (0.3175–0.4591)
LogisticRegression	0.8030(0.7790–0.8152)	0.7442 (0.7256–0.7735)	0.3843 (0.3442–0.4086)	0.7355 (0.6475–0.7714)	0.5048 (0.4518–0.5289)
RandomForest	0.7970(0.7730–0.8291)	0.8356 (0.8231–0.8580)	0.5667 (0.4759–0.6138)	0.4433 (0.3523–0.4810)	0.4975 (0.4091–0.5299)
XGBoost	0.7820(0.7464–0.7955)	0.8445 (0.8312–0.8604)	0.5885 (0.5089–0.6800)	0.2922 (0.2333–0.3571)	0.3905 (0.3269–0.4630)
SVM	0.7263(0.7168–0.7438)	0.7692 (0.7443–0.7906)	0.3787 (0.3322–0.4164)	0.5476 (0.4619–0.5952)	0.4478 (0.3890–0.4852)
MLP	0.6653(0.6430–0.7055)	0.7622 (0.7443–0.7881)	0.3687 (0.2947–0.3945)	0.4628 (0.3381–0.4714)	0.4104 (0.3163–0.4256)

16 Supplementary Table 16: Performance scores of the HRS ML model using raw data with SMOTE

Model	AUC	Accuracy	Precision	Recall	F score
EBM	0.7911(0.7636–0.8176)	0.8147 (0.8030–0.8294)	0.5774 (0.5073–0.6531)	0.2585 (0.2208–0.3182)	0.3571 (0.3126–0.4222)
LogisticRegression	0.7904(0.7632–0.8165)	0.7295 (0.7167–0.7592)	0.4059 (0.3818–0.4381)	0.6990 (0.6721–0.7727)	0.5136(0.4904–0.5545)
RandomForest	0.7850(0.7571–0.8130)	0.8182 (0.7875–0.8236)	0.5217 (0.4641–0.5582)	0.4658 (0.4416–0.5487)	0.4922 (0.4563–0.5486)
XGBoost	0.7680(0.7382–0.7961)	0.8096 (0.7985–0.8261)	0.5773 (0.4825–0.6275)	0.2637 (0.2240–0.3214)	0.3620 (0.3088–0.4198)
SVM	0.7503(0.7133–0.7818)	0.7452 (0.7289–0.7714)	0.4079 (0.3788–0.4462)	0.6161 (0.5519–0.6591)	0.4908 (0.4529–0.5277)
MLP	0.6686(0.6474–0.7135)	0.7398 (0.7212–0.7611)	0.3464 (0.3097–0.3969)	0.3564 (0.3084–0.4123)	0.3513 (0.3107–0.3988)

17 Supplementary Table 17: Performance scores of the KLoSA ML model using raw data with SMOTE

Model	AUC	Accuracy	Precision	Recall	F score
RandomForest	0.7844(0.7632–0.8126)	0.8086 (0.7927–0.8285)	0.5558 (0.5242–0.6343)	0.4568 (0.3945–0.5052)	0.5015 (0.4547–0.5547)
EBM	0.7765(0.7454–0.8065)	0.8174 (0.8016–0.8307)	0.6781 (0.5887–0.7364)	0.3230 (0.2491–0.3564)	0.4376 (0.3529–0.4717)
XGBoost	0.7749(0.7552–0.8165)	0.8046 (0.7987–0.8285)	0.6445 (0.5758–0.7305)	0.2772 (0.2353–0.3392)	0.3877 (0.3383–0.4572)
LogisticRegression	0.7594(0.7273–0.7799)	0.7221 (0.6913–0.7383)	0.4147 (0.3718–0.4320)	0.6364 (0.6055–0.7093)	0.5022 (0.4633–0.5321)
SVM	0.7358(0.7011–0.7675)	0.7193 (0.7054–0.7524)	0.4091 (0.3765–0.4464)	0.5887 (0.5329–0.6471)	0.4827 (0.4444–0.5233)
MLP	0.6711(0.6646–0.7055)	0.7112 (0.6838–0.7293)	0.3449 (0.3195–0.3960)	0.4653 (0.3978–0.5121)	0.3962 (0.3566–0.4418)

18 Supplementary Table 18: Performance scores of the MHAS ML model using raw data with SMOTE

Model	AUC	Accuracy	Precision	Recall	F score
LogisticRegression	0.7709(0.7614–0.7995)	0.7128 (0.6929–0.7267)	0.5196 (0.5019–0.5443)	0.6929 (0.6575–0.7210)	0.5939 (0.5730–0.6176)
EBM	0.7701(0.7598–0.7986)	0.7234 (0.7279–0.7556)	0.6356 (0.5893–0.6591)	0.4145 (0.3761–0.4421)	0.5018 (0.4622–0.5262)
RandomForest	0.7668(0.7570–0.7861)	0.7263 (0.7114–0.7448)	0.5494 (0.5315–0.5855)	0.5826 (0.5380–0.6040)	0.5655 (0.5382–0.5908)
XGBoost	0.7551(0.7362–0.7957)	0.7295 (0.7175–0.7475)	0.5889 (0.5616–0.6360)	0.4145 (0.3686–0.4359)	0.4865 (0.4485–0.5144)
SVM	0.7405(0.7199–0.7602)	0.7042 (0.6856–0.7198)	0.5097 (0.4930–0.5367)	0.6490 (0.6227–0.6887)	0.5710 (0.5541–0.5993)
MLP	0.7185(0.6970–0.7389)	0.6782 (0.6649–0.6998)	0.4828 (0.4647–0.5116)	0.5883 (0.5616–0.6301)	0.5304 (0.5119–0.5614)

19 Supplementary Table 19: Performance scores of the SHARE ML model using raw data with SMOTE

Model	AUC	Accuracy	Precision	Recall	F score
EBM	0.7651(0.7544–0.7962)	0.7756 (0.7696–0.7838)	0.6358 (0.6137–0.6605)	0.3555 (0.3435–0.3837)	0.4560 (0.4424–0.4825)
XGBoost	0.7630(0.7521–0.7939)	0.7693 (0.7653–0.7794)	0.6066 (0.5922–0.6371)	0.3828 (0.3543–0.3940)	0.4694 (0.4459–0.4841)
LogisticRegression	0.7602(0.7492–0.7814)	0.6998 (0.6887–0.7073)	0.4597 (0.4416–0.4644)	0.6818 (0.6612–0.6987)	0.5491 (0.5310–0.5559)
RandomForest	0.7598(0.7284–0.7709)	0.7447 (0.7369–0.7545)	0.5201 (0.5028–0.5357)	0.5392 (0.5084–0.5507)	0.5295 (0.5077–0.5405)
SVM	0.7421(0.7203–0.7641)	0.6962 (0.6898–0.7082)	0.4570 (0.4413–0.4645)	0.6616 (0.6392–0.6776)	0.5406 (0.5235–0.5498)
MLP	0.7178(0.6948–0.7397)	0.7090 (0.6921–0.7115)	0.4529 (0.4370–0.4647)	0.5803 (0.5585–0.5973)	0.5087 (0.4918–0.5209)

20 Supplementary Table 20: Indicators meaning and description

Variable Type	Variable Name	Variable Value	Handling of Variable	Definitions in Six Surveys					
				CHARLS	ELSA	HRS	KLOSA	MHAS	SHARE
**dependent variable**	depressive symptoms	None=0, Yes=1	None=0, Yes=1	CESD-10 score less than 10 indicates no depressive symptoms risk = 0	CESD-8 score less than 3 indicates no depressive symptoms risk = 0	CESD-8 score less than 3 indicates no depressive symptoms risk = 0	CESD-10 score less than 10 indicates no depressive symptoms risk = 0	CESD-9 score less than 5 indicates no depressive symptoms risk = 0	Euro-D scale score less than 4 indicates no depressive symptoms risk = 0
**independent variable**	age	50≤age<60=1, 60≤age<90=2, age≥90=3	Classified as 1,2,3	consistent	consistent	consistent	consistent	consistent	consistent
	gender	Female=0, Male=1	Two categories	consistent	consistent	consistent	consistent	consistent	consistent
	Marital status	Single, separated, divorced, widowed, married without spouse=0; married=1	Two categories	Never married, separated, divorced, widowed, married but living together = 0	Divorced, separated, never widowed=0	Never married, separated, divorced, widowed, married with absent spouse=0	Never married, separated, divorced, widowed, married but living together = 0	Never married, separated, divorced, widowed=0	Never married, separated, divorced, widowed=0
	education level	Junior high school or below=1, high school=2, higher education=3	Categorical variables	consistent	consistent	consistent	consistent	consistent	consistent
	family wealth	Quartiles, lowest quartile = 4, highest quartile = 1	quartiles	non-housing financial wealth	Total household wealth (including housing wealth)	Total household wealth (including housing wealth)	non-housing financial wealth	total household income	nonhousing wealth
**lifestyle factors**	household income	Quartiles, lowest quartile = 4, highest quartile = 1	quartiles	total household income	Household income at the couple level	couple income	total household income	couple household income	total household income
	Employment status	Unemployed=1, Retired=2, Working=3	Categorical variables	Unemployed=1, retired=2, employed =3	Unemployed/disabled/taking care of family=1, semi-retired/retired=3	Unemployment=1, retired and other status=3	Unemployed=1, retired=2, employed =3	Unemployed/disabled/out of the labor force=1, retired=3	Unemployed/permanent=1, semi-retired/homeless=3

	smoking	Never smoked = 1, former smoker = 2, current smoker = 3	Categorical variables	consistent	consistent	consistent	consistent	consistent	consistent
	drinking	Never drink alcohol = 0, current or former drinker = 1	Two categories	Not drinking now = 0, drinking now = 1	Not drinking now=1	Not drinking now = 0, drinking now = 1	Not drinking now = 0, drinking now = 1	Not drinking alcohol weekly = 0, drinking alcohol weekly or within the past 7 days = 1	Not drinking alcohol in the past 12 months = 1
**health related factors**	Self-rated health	Poor=1, Fair=2, Good/Very Good=3	Three categories	Self-assessed health, poor=1, fair=2, good/very good=3	consistent	consistent	consistent	consistent	consistent
	Activities of Daily Living (ADL)	No difficulty = 0, any difficulty = 1	Two categories	Any ADL difficulty = 1	consistent	consistent	consistent	consistent	consistent
	Instrumental Activities of Daily Living (IADL)	No difficulty = 0, any difficulty = 1	Two categories	Any IADL difficulty = 1	consistent	consistent	consistent	consistent	consistent
	Number of chronic diseases	0 species=1, 1 species=2, ≥2 species=3	Three categories	Number of self-reported chronic diseases, divided into 0, 1, and ≥2	consistent	consistent	consistent	consistent	consistent
	pain distress	No pain = 0, pain = 1	Two categories	Self-reported pain, yes=1	consistent	consistent	consistent	consistent	consistent
	word memory	standardized score	continuous variable	Word Memory Test Score, Standardized	consistent	consistent	consistent	consistent	consistent

date targeting	0=All wrong, 1=One item correct, 2=Two items correct, 3=All correct	Orderly classification	Correct reporting quantity for year, month and day	consistent	consistent	consistent	consistent	consistent
executive function	0-5 times correct	Orderly classification	Continuously subtract 7 to test the number of correct times	consistent	consistent	consistent	consistent	consistent

21 Supplementary Table 21: Hyperparameter settings for the optimal model in each of the six surveys

Six Surveys	Best Model	Parameter setting
CHARLS	EBM	max_bins=256, outer_bags=100, inner_bags=0, learning_rate=0.01
ELSA	LogisticRegression	penalty=l2, C=1.0, solver=saga, max_iter=1000
HRS	EBM	max_bins=256, outer_bags=100, inner_bags=0, learning_rate=0.01
KLOSA	EBM	max_bins=256, outer_bags=100, inner_bags=0, learning_rate=0.01
MHAS	EBM	max_bins=256, outer_bags=100, inner_bags=0, learning_rate=0.01
SHARE	EBM	max_bins=256, outer_bags=100, inner_bags=0, learning_rate=0.01

22 Supplementary Figure 1: Flow chart of participants selected in the CHARLS

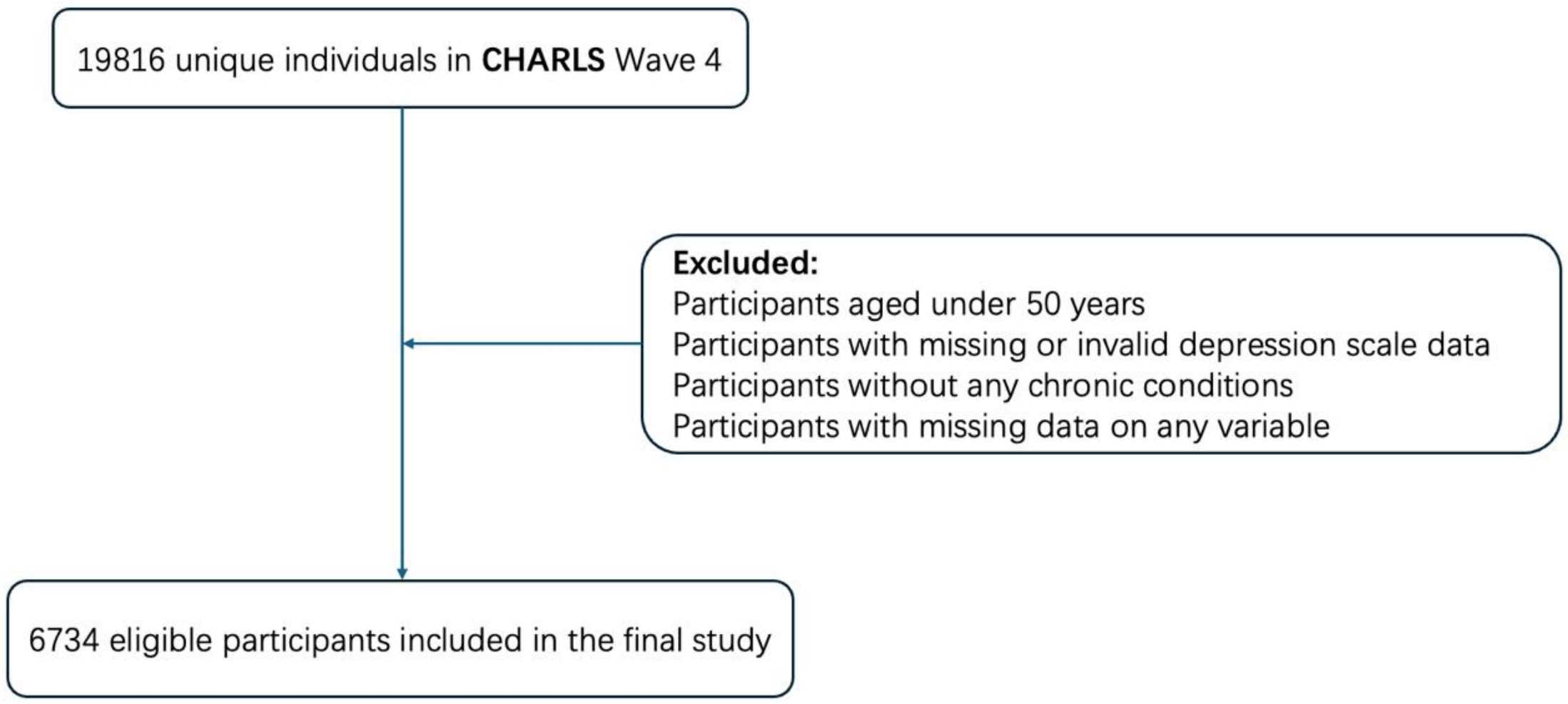

23 Supplementary Figure 2: Flow chart of participants selected in the ELSA

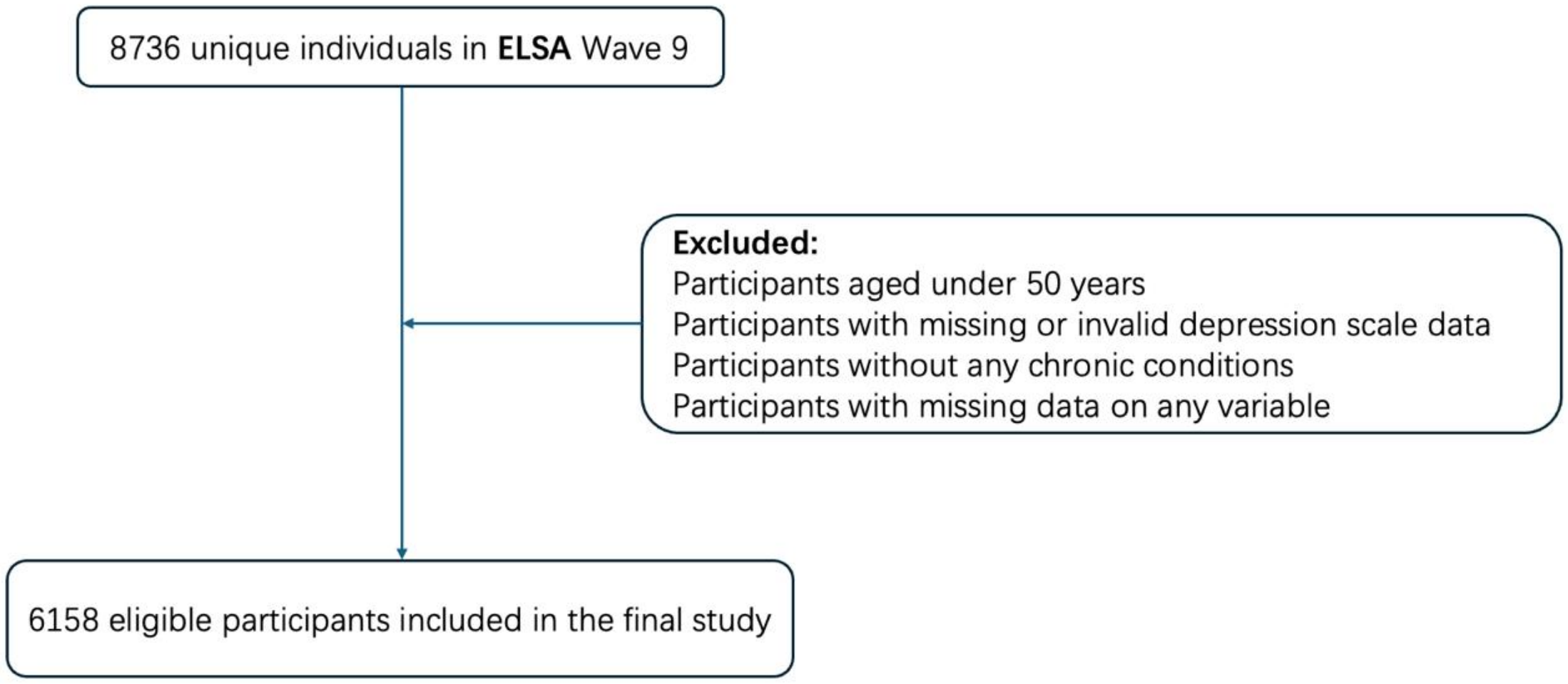

24 Supplementary Figure 3: Flow chart of participants selected in the HRS

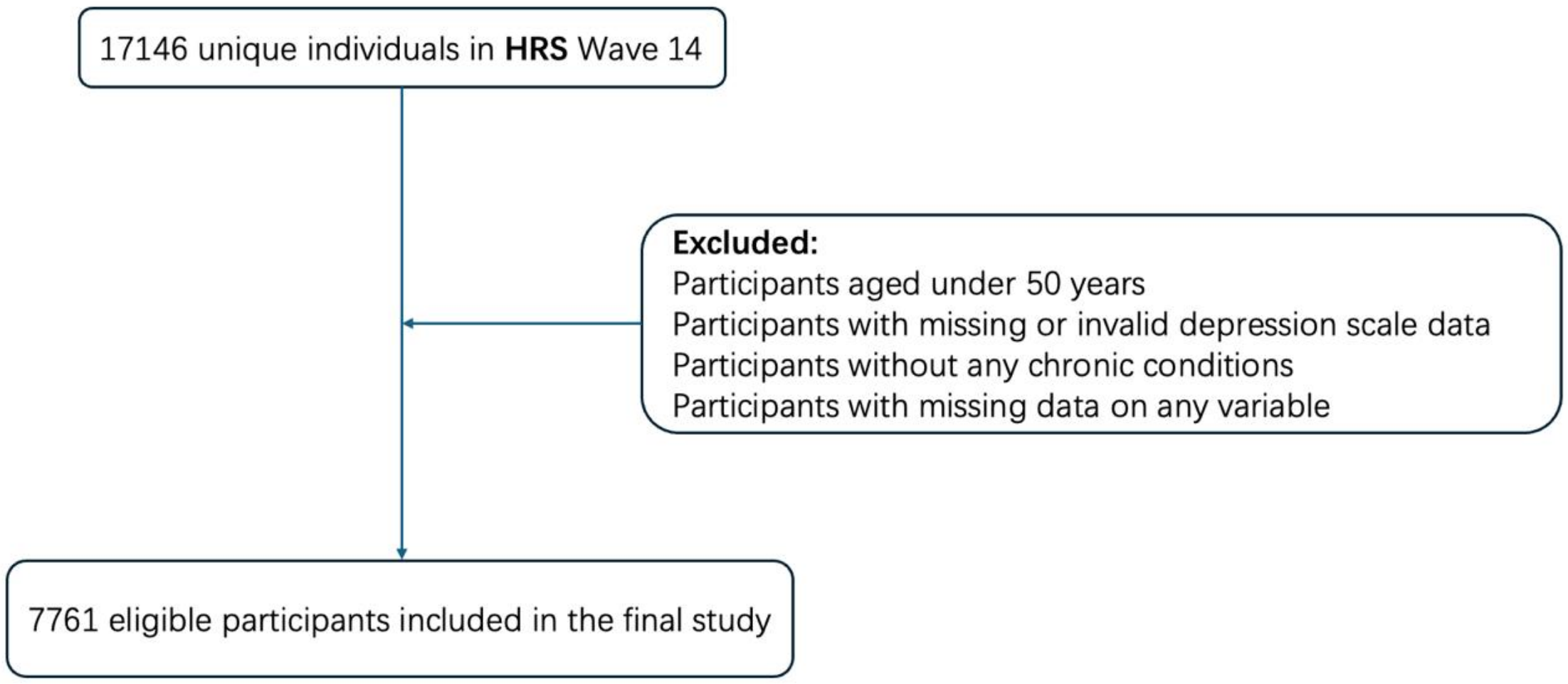

25 Supplementary Figure 4: Flow chart of participants selected in the KLoSA

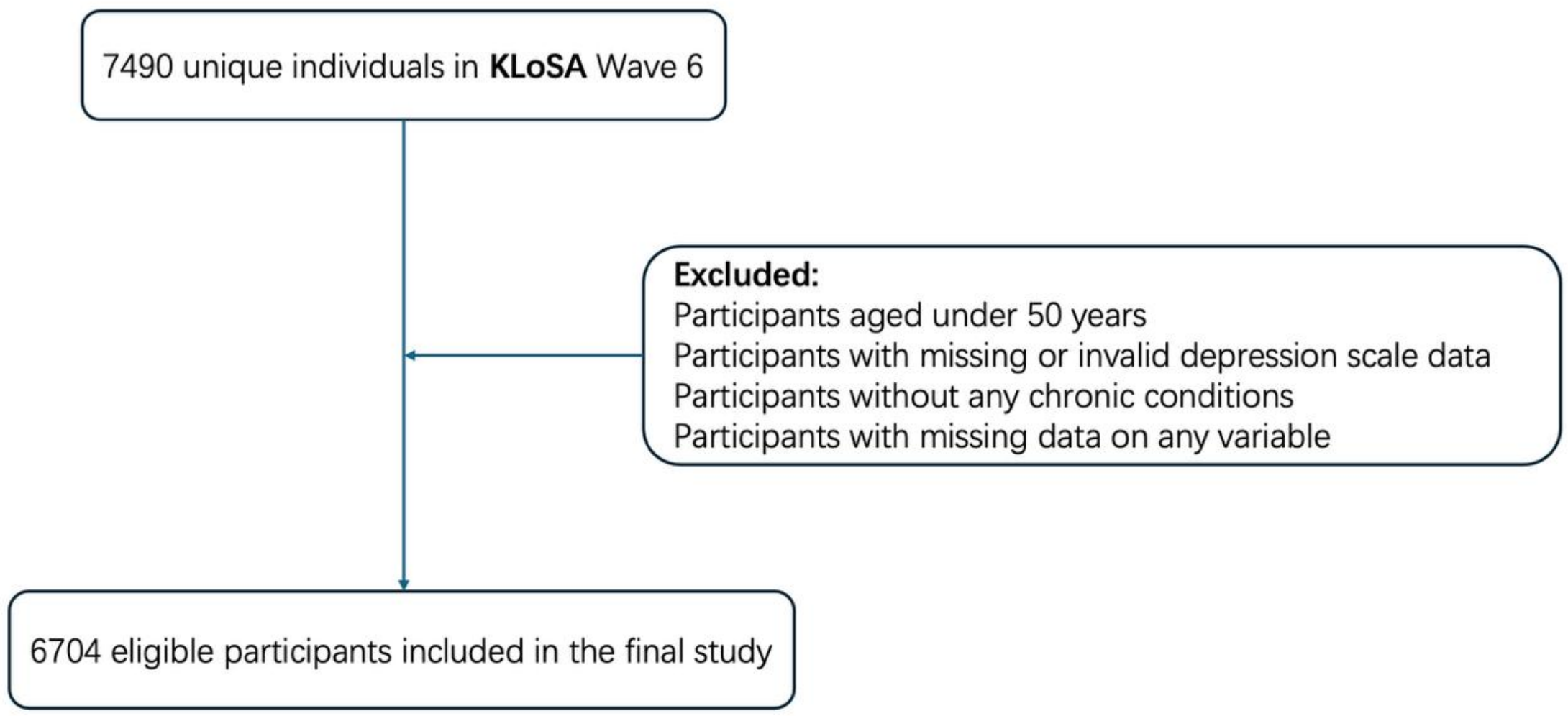

26 Supplementary Figure 5: Flow chart of participants selected in the MHAS

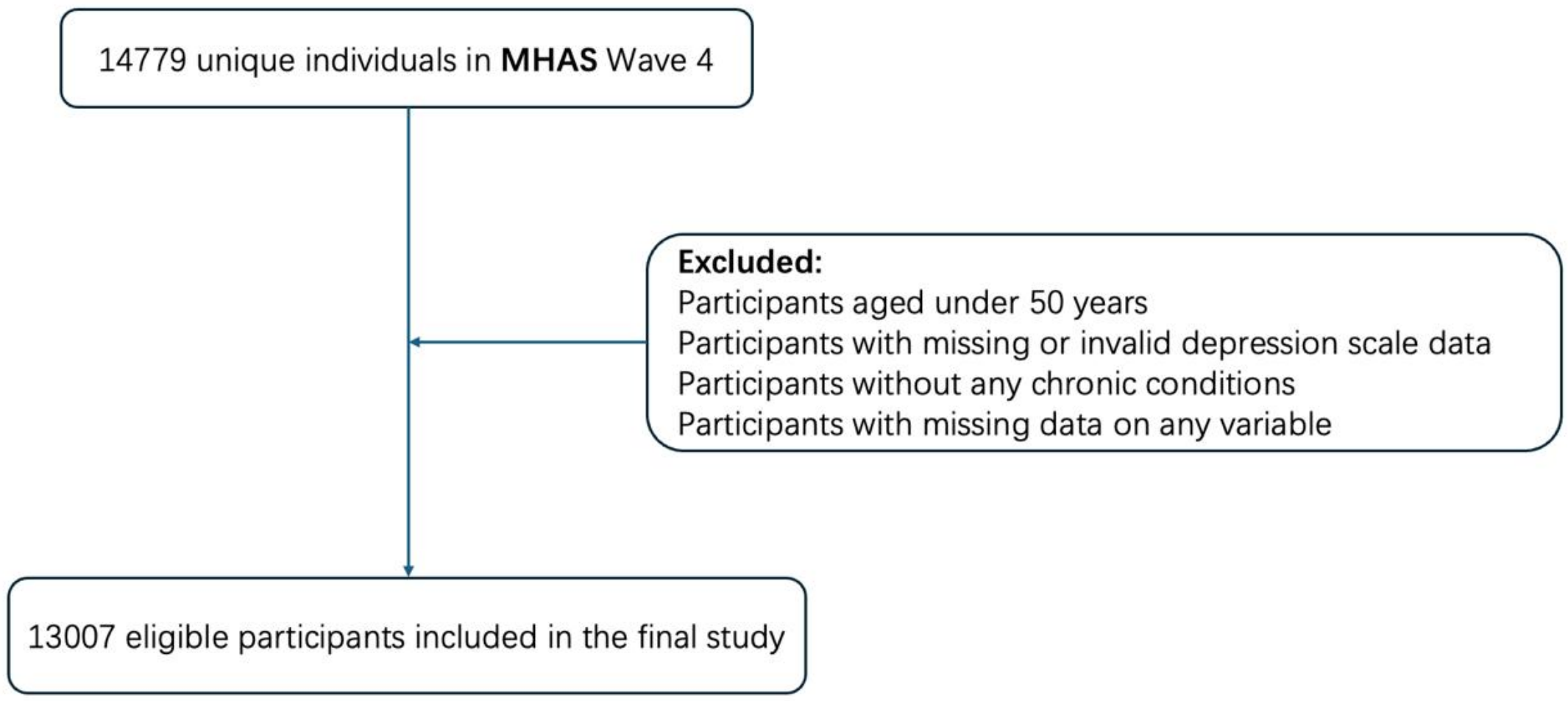

27 Supplementary Figure 6: Flow chart of participants selected in the SHARE

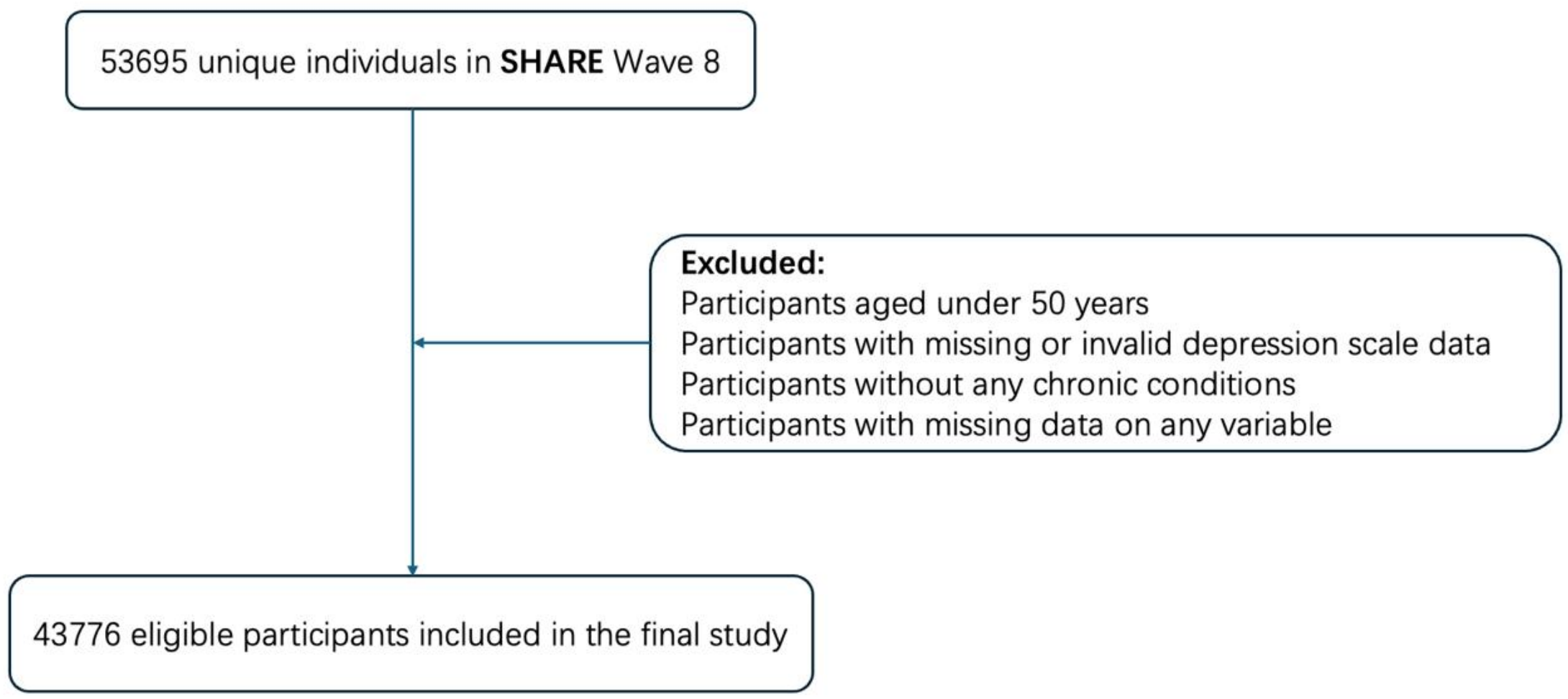

28 Supplementary Figure 7: Cross-national comparison of mean contributions for key predictors across six cohorts

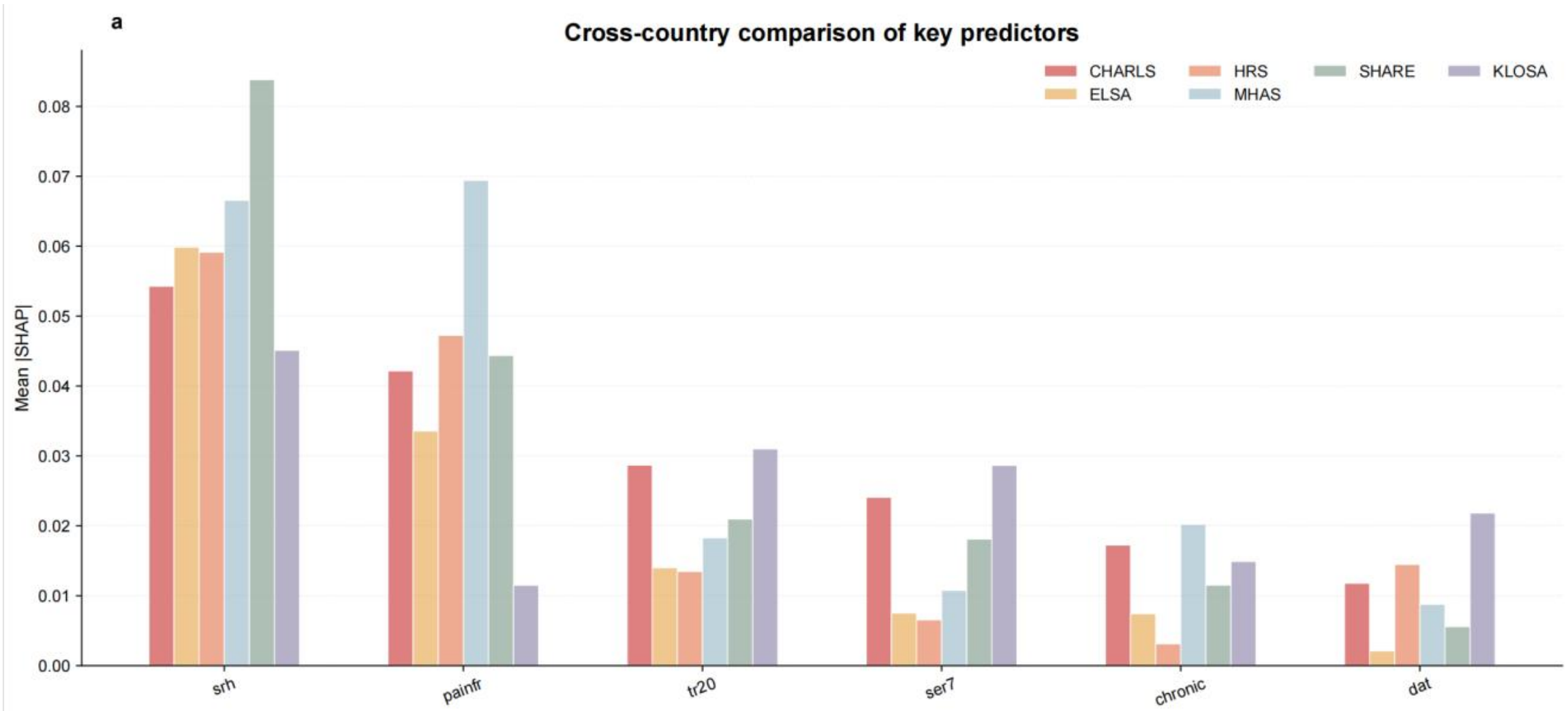

Supplement: Supplementary file 1 [file DataSheet1.pdf]
